# Antisense Oligonucleotides: Technological Advances, Clinical Progress, and Expanding Therapeutic Frontiers

**DOI:** 10.3390/pharmaceutics18040446

**Published:** 2026-04-04

**Authors:** Liping Xu, Huaqun Zhang, Bingchen Jiang, Yuanying Jiang, Hui Lu

**Affiliations:** 1 Department of Pharmacy, Shanghai Tenth People’s Hospital, School of Medicine, Tongji University, Shanghai 200331, China; 2 Department of Chemistry and Biochemistry, The Ohio State University, Columbus, OH 43210, USA; 3 Shanghai Municipal Hospital of Traditional Chinese Medicine, Shanghai 200071, China; 4 Key Laboratory of Pathogen-Host Interaction, Ministry of Education, School of Medicine, Tongji University, Shanghai 200331, China

**Keywords:** antisense oligonucleotides, RNA-targeted therapeutics, chemical modification, precision medicine

## Abstract

Antisense oligonucleotides (ASOs) are emerging therapeutic agents that modulate gene expression at the RNA level, offering distinct therapeutic advantages over conventional small-molecule drugs and biologics. By directly targeting RNA, ASOs expand the spectrum of druggable targets to include those previously considered “undruggable”, and enable shorter development timelines with improved research and development efficiency. These attributes position ASOs as a highly promising platform for precision and personalized medicine. Recent advances in chemical modification strategies and delivery technologies have markedly accelerated their clinical translation. This review systematically examines the technological evolution of ASO therapeutics, detailing their mechanisms of action, key chemical modification strategies, and advanced delivery systems. It also provides a comprehensive overview of the current global clinical landscape, including approved drugs, discontinued candidates, and ongoing clinical trials. Finally, this review discusses the major challenges facing the field and outlines future directions, with the aim of informing subsequent basic research and clinical development efforts.

## 1. Introduction

RNA functions as a central mediator of cellular information flow and gene regulation. Increasing evidence suggests that both messenger RNA (mRNA) and non-coding RNA (ncRNA) contain highly structured and functionally critical elements, and that aberrations within these elements are closely linked to the pathogenesis of numerous human diseases [1]. Notably, only approximately 1.5% of the human genome encodes proteins, and up to 80% of protein-coding targets are considered “undruggable” by conventional therapeutic methods, posing a substantial challenge to drug discovery [2]. RNA-targeting therapeutics have therefore emerged as a transformative strategy, enabling access to previously inaccessible regulatory pathways and expanding the landscape of druggable targets.

Among diverse RNA-targeting strategies, oligonucleotide therapeutics have achieved remarkable clinical progress, owing to their broad target accessibility, relatively streamlined development paradigms, and controllable manufacturing costs. This class includes antisense oligonucleotides (ASOs), small interfering RNAs (siRNAs), microRNAs (miRNAs), small activating RNAs (saRNAs), and aptamers [3]. ASOs are typically single-stranded oligonucleotides that bind complementary pre-mRNA or mRNA and act either by RNase H1-mediated degradation or by steric modulation of splicing or translation [4]. By contrast, siRNAs are short double-stranded RNAs that are loaded into the AGO2-containing RNA-induced silencing complex and mainly induce cleavage of cytoplasmic mRNA [3]; miRNA therapeutics are generally developed as mimics or inhibitors and often modulate broader gene networks rather than a single transcript [5]; saRNAs are short double-stranded RNAs that target promoter-associated sequences to activate gene transcription [6]; and aptamers are structured single-stranded oligonucleotides that bind proteins or other molecular targets in an antibody-like manner rather than through antisense pairing to RNA [7]. Currently, ASOs represent the most established modality among approved oligonucleotide drugs, with 14 marketed products (including subsequently withdrawn agents), outnumbering siRNAs (8) and aptamers (2), underscoring their relative maturity and leadership in clinical translation [8,9]. This relative maturity partly reflects the mechanistic versatility of ASOs, which can act on both nuclear and cytoplasmic RNA and support transcript knockdown, as well as splice modulation. Continuous technological innovation, particularly iterative advances in chemical modifications and delivery platforms, has been fundamental to this success [3]. These innovations have addressed key barriers to clinical translation, including limited in vivo stability, inefficient cellular uptake, and off-target toxicity. Moreover, improved translational strategies and growing clinical validation have enabled ASOs to expand beyond rare genetic disorders into broader chronic indications.

This review offers a systematic overview of the technological evolution of ASOs, tracing their progression from conceptual inception to contemporary clinical applications. It outlines the core mechanisms underlying ASO-mediated gene regulation, summarizes major chemical modification strategies and advanced delivery systems, and analyzes the global clinical translation landscape, including approved drugs, discontinued candidates, and ongoing clinical trials. Finally, it discusses the principal challenges facing the field and highlights future development directions, with the aim of informing both fundamental research and the advancement of next-generation clinical candidates.

## 2. Historical Development and Major Inflection Points of ASOs

**Figure 1:**
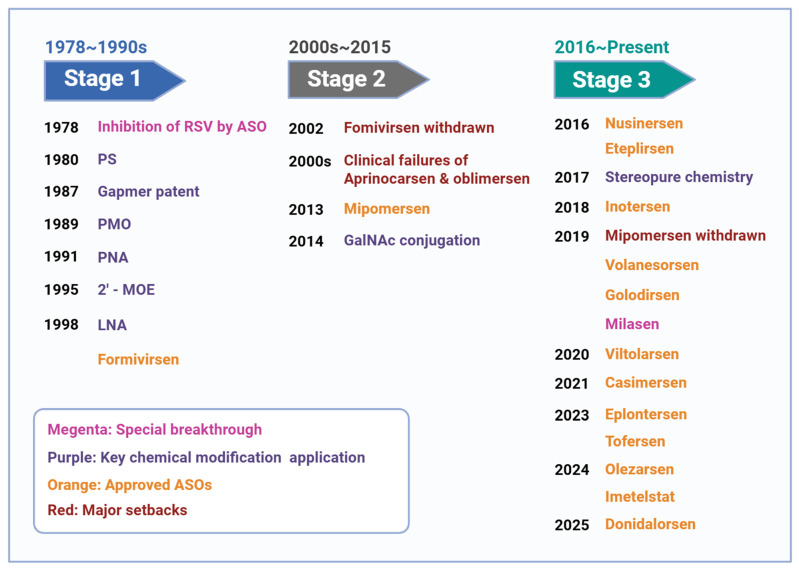
Historical development and major inflection points of ASO therapeutics. The development of ASO therapeutics has proceeded through three broad phases: foundational proof-of-concept and first-generation chemistry, a subsequent period of clinical setbacks and technological redesign, and a modern phase of clinical expansion supported by improved chemistry and delivery. Created in BioRender. Xu, A. (2026) https://BioRender.com/r3hx2m7, accessed on 31 March 2026.

The historical development of ASO therapeutics is best understood as a sequence of distinct scientific, technological, and clinical inflection points rather than as a simple linear progression (Figure 1). Early proof-of-concept validation was established by foundational sequence-specific antisense studies and the emergence of key chemistries such as phosphorothioate backbones, 2′-MOE modification, and gapmer design. This was followed by a period of major setbacks in the early 2000s, including the withdrawal of fomivirsen and multiple late-stage failures in oncology, which exposed persistent limitations in delivery, efficacy, and safety. The field subsequently entered a more productive phase driven by improved chemistry, GalNAc-based targeted delivery, and better-matched clinical indications, culminating in successive approvals across neurological, metabolic, and more recently oncological diseases.

### 2.1. Foundational Proof-of-Concept and First-Generation Chemistry (1978–1999)

In the context of RNA-based therapeutics, ASOs are synthetic, single-stranded nucleic acid analogs, typically ranging from 12 to 30 nucleotides in length, that selectively recognize and bind complementary RNA targets through Watson–Crick base pairing, thereby modulating gene expression [10]. The conceptual foundation of the field was established in 1978, when Zamecnik and Stephenson showed that a sequence-complementary oligonucleotide directed against Rous sarcoma virus 35S RNA could inhibit viral replication in cultured cells [11,12].

Over the following two decades, first-generation chemistries were introduced to improve the poor metabolic stability and pharmacokinetic limitations of unmodified oligonucleotides, most notably through phosphorothioate (PS) backbone substitution [13]. A major clinical milestone during this period was the 1998 U.S. Food and Drug Administration (FDA) approval of fomivirsen (Vitravene) for cytomegalovirus retinitis in patients with AIDS [14]. However, fomivirsen required local intravitreal administration and did not solve the broader challenges of systemic exposure, potency, and tolerability [15]. Its later withdrawal, largely because the widespread adoption of combination antiretroviral therapy markedly reduced the incidence of cytomegalovirus retinitis and thus the clinical need for the drug [16], nevertheless marked both the first clinical validation of antisense pharmacology and a reminder of the limitations of first-generation ASOs, highlighting the need for more advanced chemistry and delivery strategies.

### 2.2. Clinical Setbacks and Second-Generation Redesign (2000–2015)

At the beginning of the 21st century, the ASO field entered a period of major clinical and technological reassessment. First-generation ASOs were often limited by insufficient target affinity, non-specific immune activation, unfavorable protein interactions, and dose-limiting toxicities, and several late-stage clinical programs failed to show adequate therapeutic benefit [17,18]. This period can therefore be viewed as a translational bottleneck rather than simply a temporary stagnation. A major turning point came with the development of second-generation chemistries, particularly 2′-O-methoxyethyl (2′-MOE), together with the widespread adoption of the chimeric “gapmer” design strategy [19,20]. Gapmers contain a central DNA “gap” flanked by chemically modified nucleotides, and the modified wings improve target affinity and nuclease resistance, whereas the DNA core preserves the ability to recruit RNase H1 after hybridization to target RNA, resulting in sequence-specific RNA cleavage [19,21]. The 2013 FDA approval of mipomersen (Kynamro) for the treatment of homozygous familial hypercholesterolemia provided proof of concept that systemically administered ASOs could reduce liver-derived target proteins in humans, thereby reigniting enthusiasm for ASO development [22]. Although mipomersen later faced commercial and safety-related setbacks, including injection-site reactions and hepatotoxicity, it demonstrated that second-generation ASO chemistry could support clinically meaningful systemic target knockdown [23]. These concerns, in turn, catalyzed advances in precision engineering, including N-acetylgalactosamine (GalNAc)-mediated liver targeting, stereopure (chiral) synthesis, and improved control of ASO–protein interactions [24,25]. In addition, the regulatory pathway established for mipomersen, including orphan drug designation and risk evaluation and mitigation strategy management measures, provided an important operational framework for subsequent ASO development [26].

### 2.3. Clinical Expansion and Platform Maturation (2016–Present)

From 2016 onward, ASO development shifted from proof-of-principle to broader clinical expansion. Continued refinement of chemistry, such as locked nucleic acid (LNA) and constrained ethyl (cEt) containing designs, alongside with major advances in tissue-targeted delivery, markedly improved potency and tolerability [20,25]. A transformative milestone was the 2016 approval of nusinersen (Spinraza), which validated splice modulation as a therapeutic mechanism and showed that intrathecal delivery could successfully address central nervous system disease [27]. This success restored confidence in nucleic acid therapeutics and helped reposition ASOs as a viable drug platform rather than a niche experimental modality. In parallel, GalNAc conjugation reshaped hepatic delivery by enabling more efficient receptor-mediated uptake and lower effective doses, contributing to the development of newer liver-directed ASOs such as eplontersen and olezarsen [24,28,29]. More recent approvals, including tofersen (Qalsody) for SOD1-associated amyotrophic lateral sclerosis and imetelstat (Rytelo) as an oligonucleotide telomerase inhibitor in oncology, further illustrate the diversification of ASO mechanisms and indications beyond early orphan disease applications [30,31]. Collectively, these advances indicate that the modern ASO era is defined not simply by increased drug approvals, but by a more mature integration of chemistry, delivery, disease selection, and regulatory strategy.

## 3. Mechanisms of Action

**Figure 2:**
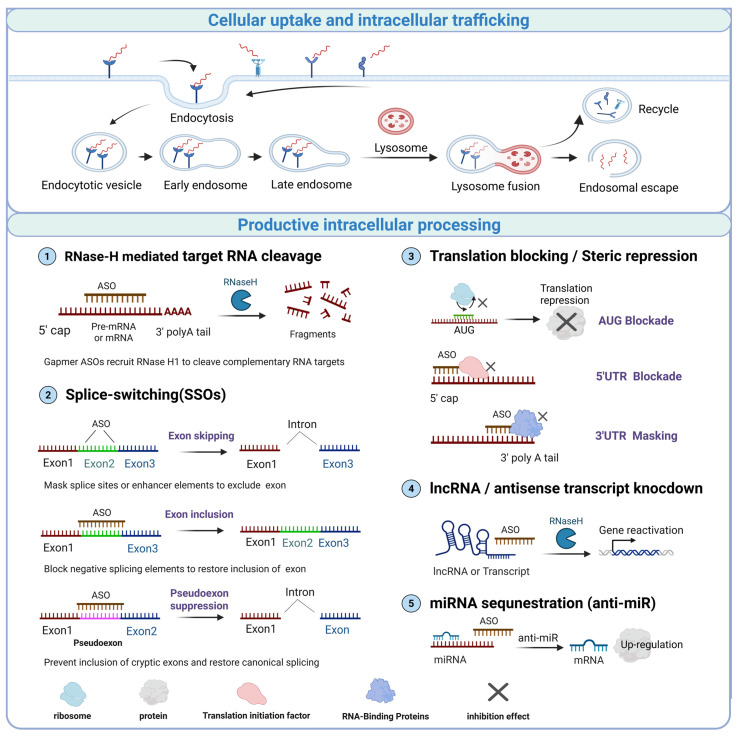
Major mechanism of action of antisense oligonucleotides (ASOs). ASOs are internalized into target cells through endocytosis, traverse the endocytotic pathway, and undergo endosomal escape to reach the cytoplasm or nucleus. Productively delivered ASOs exert their effects through multiple intracellular mechanisms, including RNase H-mediated target RNA cleavage, splice switching (exon skipping, exon inclusion, and pseudoexon suppression), translation blocking or steric repression, lncRNA or antisense transcript knockdown, and miRNA sequestration (anti-miR). Created in BioRender. Xu, A. (2026) https://BioRender.com/cmvf19h, accessed on 31 March 2026.

ASOs modulate gene expression through sequence-specific hybridization with target RNAs (Figure 2). Their action process can be conceptualized into three stages: pre-hybridization, hybridization, and post-hybridization.

### 3.1. Pre-Hybridization: Cellular Uptake and Trafficking

Upon entering the tissue, ASOs can be passively internalized through gymnosis; however, cellular uptake predominantly relies on receptor-mediated endocytosis [32]. Cell surface receptors, such as stabilin-1 and stabilin-2, recognize and internalize ASOs via clathrin-dependent pathways, serving as a major in vivo entry route [33,34]. In hepatocytes, the asialoglycoprotein receptor exhibits high endocytic efficiency; multivalent GalNAc ligands promote synergistic receptor engagement by simultaneously or rapidly rebinding multiple carbohydrate-recognition sites on ASGPR, which increases avidity and facilitates efficient ASO endocytosis [35].

Following endocytosis, ASOs traffic from early endosomes to late endosomes or multivesicular bodies. Only a small fraction can successfully escape into the cytoplasm or nucleus, where they exert their biological activity, making endosomal escape the principal rate-limiting step for therapeutic efficacy [36]. Intracellularly, ASOs form dynamic complexes with nucleic acid-binding proteins, including La/SSB, NPM1, P54nrb/NONO and PSF/SFPQ, which can influence their subcellular distribution, nuclear accumulation, and intracellular retention [13,37,38].

The chemical structure of ASOs determines their protein-binding properties, subcellular localization, and applicable administration routes [37]. Fully PS backbones enhance reversible binding to plasma proteins, increase nuclease resistance, prolong circulating half-life, and promote accumulation in highly perfused tissues such as the liver and kidneys following intravenous administration [39]. Conversely, mixed backbones, chimeric structures composed of phosphorothioate and phosphodiester linkages, reduce protein-binding affinity and accelerate renal clearance, making them appropriate for local administration settings where minimal systemic exposure is desired [40]. Chiral phosphorus backbones further improve target RNA-binding specificity through defined spatial configuration, thereby reducing off-target effects and informing the selection of optimal administration routes [41].

### 3.2. Hybridization: Target RNA Recognition

Following successful intracellular trafficking to the appropriate subcellular compartments, ASOs initiate sequence-specific molecular recognition and hybridization with their target RNA [42]. To achieve effective binding, they must overcome steric hindrance imposed by complex RNA structures, such as hairpins and pseudoknots, and dynamically compete with endogenous RNA-binding proteins already associated with the transcript [43]. These proteins include spliceosomal components that regulate pre-mRNA splicing, heterogeneous nuclear ribonucleoproteins responsible for RNA transport and processing, as well as cytoplasmic mRNA-binding proteins involved in mRNA stability and translation, which can also influence target-site accessibility on mature transcripts, in addition to ribosomes engaged in translation [44,45]. The spatial accessibility of the target site, along with the hybridization kinetics, are key efficacy determinants. These factors govern binding stability, target engagement efficiency, and ultimately the magnitude and durability of the pharmacological response [46].

### 3.3. Post-Hybridization: Functional Modulation of Target RNA

Upon formation of a stable ASO-target RNA complex, ASOs exert their effects through distinct mechanistic pathways defined by how they modulate target RNA function. A major class operates via RNase H1-dependent cleavage. In this mechanism, ASOs form DNA/RNA heteroduplexes with the target transcript and recruit the endogenous endonuclease RNase H1, which catalyzes site-specific cleavage of phosphodiester bonds within the RNA strand. This process leads to degradation of pathogenic transcripts and suppression of pathogenic protein synthesis [47]. Representative clinically validated examples include mipomersen, which degrades APOB mRNA to inhibit apoB-100 synthesis and reduce circulating apoB-containing lipoproteins and LDL-C, as well as inotersen and eplontersen, which degrade TTR mRNA to suppress transthyretin production and thereby reduce pathogenic TTR burden [22]. Because RNase H1 is localized in both the cytoplasm and nucleus, this pathway enables the degradation of mature cytoplasmic mRNAs, as well as nuclear-retained pre-mRNAs and immature transcripts, thereby broadening the spectrum of targetable RNAs [44].

In contrast, RNase H1-independent ASOs function primarily through steric hindrance rather than target cleavage. By binding specific RNA sequences, these ASOs physically block the access of regulatory proteins, spliceosomal components, or ribosomes, thereby modulating RNA processing or translation [48]. One major subclass of this category is splice-switching oligonucleotides (SSOs), namely steric-blocking ASOs that bind pre-mRNA and redirect spliceosome assembly. At the molecular level, SSOs can mask 5′ or 3′ splice sites, branch points, polypyrimidine tracts, or exonic/intronic splicing enhancers or silencers, thereby promoting exon skipping or inclusion, suppressing pseudoexon incorporation, or preventing cryptic splice-site usage [48,49]. Within the nucleus, splice-switching ASOs target exon–intron junctions or splicing regulatory elements to modulate alternative splicing. For example, in Duchenne muscular dystrophy, exon-skipping SSOs restore the translational reading frame and enable production of internally truncated but partially functional dystrophin and thereby supporting disease-modifying benefit [49]. Conversely, other SSOs promote exon inclusion; for example, nusinersen (Spinraza) targets SMN2 pre-mRNA to enhance exon 7 inclusion, thereby increasing the production of functional SMN protein and improving motor outcomes and survival in spinal muscular atrophy [50]. Outside the nucleus, steric-blocking ASOs can also act in the cytoplasm by binding regulatory regions such as the 5′ untranslated region or upstream open reading frames, thereby either relieving inhibitory elements to enhance translation or obstructing initiation to reduce protein expression [51]. This steric mode of action can further extend to non-coding RNAs, including miRNAs and long non-coding RNAs, by interfering with their processing, maturation, or functional interactions rather than through direct RNase H1-mediated degradation.

Beyond these two classic mechanisms, several emerging ASO modalities are under active investigation. One notable example is ADAR-recruiting RNA editing, in which antisense oligonucleotides recruit endogenous adenosine deaminases acting on RNA (ADARs) to direct site-specific adenosine-to-inosine (A-to-I) conversion on target transcripts. This strategy, sometimes termed ADAR-mediated editing oligonucleotides or AIMers in recent reports, enables transient correction of selected pathogenic variants at the RNA level without altering genomic DNA [52]. Additionally, ASOs can form triplex structures with DNA or RNA duplexes to inhibit transcription [53], or mask RNA-binding protein recognition motifs, thereby modulating RNA splicing, stability, and translation efficiency [54]. Collectively, these expanding mechanistic modalities significantly broaden the therapeutic scope of ASOs in gene regulation and precision therapeutics.

## 4. Chemical Modifications

**Figure 3:**
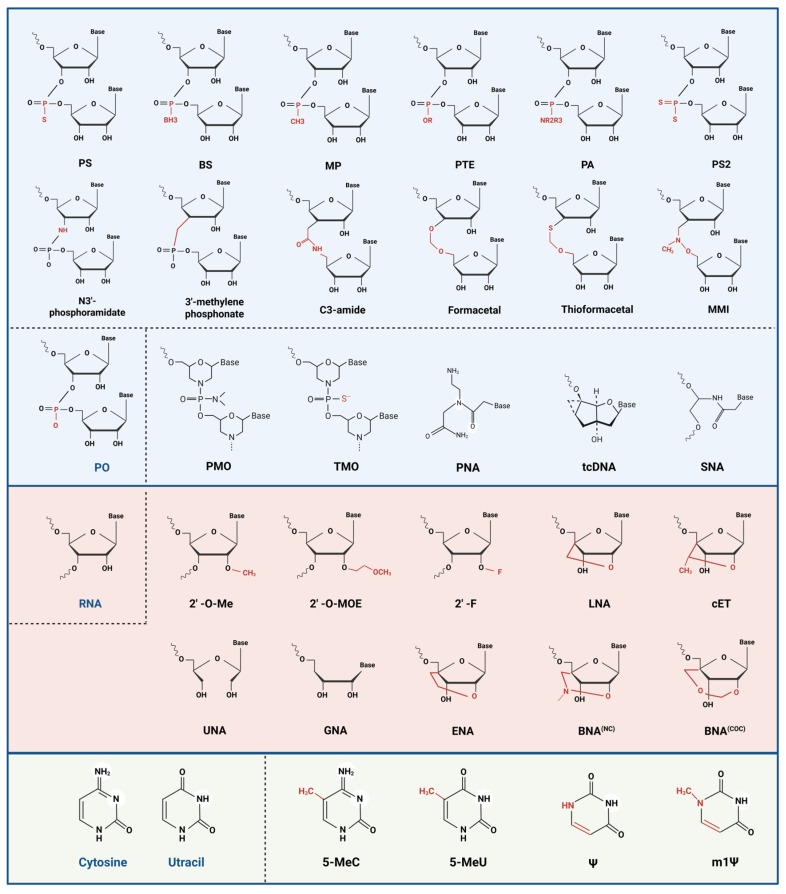
Representative chemical structures of major ASO chemical modifications. Representative chemical modifications organized into backbone, ribose, and base modifications, native or canonical reference structures are included where appropriate to facilitate structural comparison. This figure is intended to provide a structural overview of the major ASO-relevant modifications discussed in Section 4, whereas Table 1 summarizes their classification, approximate time of first report, and principal functional purposes. Created in BioRender. Xu, A. (2026) https://BioRender.com/psapmx6, accessed on 31 March 2026.

Chemical modification has been a defining feature of ASO development, as it directly influences molecular stability, hybridization properties, pharmacokinetics, tolerability, biological activity, and, in some cases, the mechanism of action [55]. To provide a clearer framework, Table 1 summarizes the major chemical modifications used in ASO design, including their classification, approximate time of first report, and principal functional purposes. Figure 3, by contrast, illustrates the representative chemical structures of these modifications. Thus, while the table provides a functional and historical summary, the figure offers a structural view of the same modification landscape. On this basis, the discussion below is organized into backbone, ribose, and base modifications.

### 4.1. Backbone Modifications

Backbone modifications are central to improving metabolic stability, minimizing immunogenicity, and optimizing tissue distribution, with phosphodiester (PO) linkage modification as the foundational strategy [13,47]. Among these, PS substitution, where a non-bridging phosphate oxygen is replaced by sulfur, is the most clinically established backbone modification [13]. This sulfur substitution increases resistance to nuclease-mediated cleavage and alters interactions with plasma and cellular proteins, thereby prolonging circulation and influencing tissue distribution, while still allowing recruitment of RNase H1 after hybridization to target RNA [56]. Accordingly, the majority of approved ASOs incorporate PS linkages. The “gapmer” structure further optimizes the therapeutic index by combining a central DNA-like “gap,” which supports RNase H1-mediated cleavage, with chemically modified “wings” that enhance affinity and stability [48]. Moreover, stereoselective synthesis controlling the chirality (Sp or Rp) of PS linkages reduces backbone heterogeneity, and can modulate protein binding, as well as RNase H1 cleavage patterns, to mitigate non-specific interactions and enhances efficacy [41,57]. Emerging non-natural linkages, such as mesyl-phosphoramidate (MsPA), phosphoryl-guanidine (PG), and boranophosphate (PB), further tune backbone charge density and local conformation, with the aim of preserving target engagement while reducing non-specific immune or protein interactions [58,59].

Another alternative strategy involves modifying the topological framework of the oligonucleotide [12]. Phosphorodiamidate morpholino oligomers (PMOs) replace the ribose ring with a morpholine moiety linked via neutral phosphorodiamidate bonds, substantially reducing non-specific protein interactions [20,49]. Since the neutral backbone shows low protein binding and does not support RNase H recruitment, PMOs act primarily through steric blockade and are therefore particularly suited to splice-switching applications, as exemplified by the approved PMO drug, eteplirsen (Exondys 51), for Duchenne muscular dystrophy [60]. Thiophosphoramidate morpholinos (TMOs) further improve stability through sulfur or nitrogen modifications [61]. Peptide nucleic acids (PNAs) use a neutral pseudopeptide backbone that eliminates electrostatic repulsion, thereby strengthening target-binding affinity, although cellular uptake and solubility remain limiting challenges [62]. Similarly, serinol nucleic acids (SNAs), in which ribose is replaced by serinol, enable hybridization with various chiral oligonucleotides, offering additional opportunities to optimize the stability and sequence specificity [63,64].

### 4.2. Sugar Modifications

Sugar modifications, particularly at the 2′ position of the ribose ring or through conformational locking, substantially enhance target affinity, improve stability, and reduce toxicity [65]. Classical non-bridged 2′ modifications, such as 2′-O-methyl (2′-OMe), 2′-O-methoxyethyl (2′-MOE), and 2′-fluoro (2′-F), favor a C3′-endo sugar pucker, thereby strengthening hybridization to complementary mRNA sequences [66]. Among these, 2′-MOE, a representative of second-generation chemistry, confers enhanced nuclease stability and attenuates immunogenicity [67]. The 2′-OMe modification is synthetically accessible and well tolerated, whereas 2′-F further increases binding affinity with minimal steric hindrance, albeit with potential concerns regarding metabolite-associated toxicity [68]. The clinical success of nusinersen (Spinraza), an 18-mer PS backbone ASO uniformly modified with 2′-MOE, exemplifies the impact of sugar chemistry optimization, enabling high-affinity binding to SMN2 pre-mRNA and pioneering splicing–modulating therapy for spinal muscular atrophy [69]. Additionally, the 2′-O-[2-(N-methylcarbamoyl) ethyl] (2′-MCE) modification demonstrates comparable activity to 2′-MOE with reduced hepatotoxicity, positioning it as a promising next-generation 2′-O-alkyl alternative [70].

Conformationally locked bridged nucleic acids (BNAs), which establish a 2′-4′ chemical bridge, have been developed to enhance ASO hybridization affinity and structural rigidity by preorganizing the sugar into a C3′-endo conformation, thereby reducing backbone flexibility and the entropic cost of duplex formation with complementary RNA [71]. Locked nucleic acids (LNAs), characterized by a 2′-O,4′-C-methylene bridge, increase the melting temperature by approximately 3–8 °C per monomer [72]. The introduction of a methyl group to LNAs, forming constrained ethyl (cEt) derivatives, often preserves high affinity while reducing the hepatotoxicity observed with some LNA-based designs, thereby improving the therapeutic index [73]. Ongoing efforts in BNA chemistry aim to retain the benefits of conformational locking while further reducing toxicity and optimizing tissue distribution; representative examples include BNAP-AEO, which has shown reduced acute neurotoxicity, and cycloalkane-incorporated BNAs, which improve nuclease stability and may reduce the need for extensive PS modification while maintaining efficacy [71,74].

For ribose-deficient backbones systems such as PMO and PNAs, limited membrane permeability and suboptimal solubility remain the key challenges, which are being addressed through modifications of side-chains, linkages, or terminals [47]. For example, arginine-rich cell-penetrating peptides promote endocytosis [75], cationic linkers create “charge-chimeric” PMOs to enhance muscle uptake [76], and lipid or GalNAc conjugates enable efficient tissue-selective biodistribution: lipid conjugates can alter plasma protein/lipoprotein association and thereby bias tissue exposure, whereas GalNAc ligands bind the asialoglycoprotein receptor on hepatocytes to promote receptor-mediated uptake and liver-selective accumulation [6,77].

### 4.3. Nucleobase Modification

Unmodified CpG motifs, which are cytosine–guanine dinucleotides, activate Toll-like receptor 9 (TLR9), leading to severe influenza-like symptoms and inflammatory responses in early candidate ASOs [78]. The modification of CpG motifs with 5-methylcytosine (5-MeC) effectively mimics endogenous DNA methylation, thereby reducing TLR9 recognition and preventing innate immune activation without substantially altering heteroduplex geometry [65,79]. This kind of modification is frequently employed in current clinical ASOs.

To develop shorter and more potent ASOs, heterocyclic bases are engineered to introduce additional hydrogen bonds or enhance stacking interactions. For instance, C5-propynyl substitutions (C5-propynyl-C/U) strengthen π–π stacking and stabilize duplex formation, thereby increasing the melting temperature [79,80]. Additionally, the G-clamp, a tricyclic cytosine analog, forms four hydrogen bonds with guanine, and also provides favorable stacking interactions, enabling higher-affinity target recognition and supporting the design of shorter ASOs (10~12 nucleotides) than the conventional ~20-nucleotide format [81,82].

## 5. Delivery Strategies

**Figure 4:**
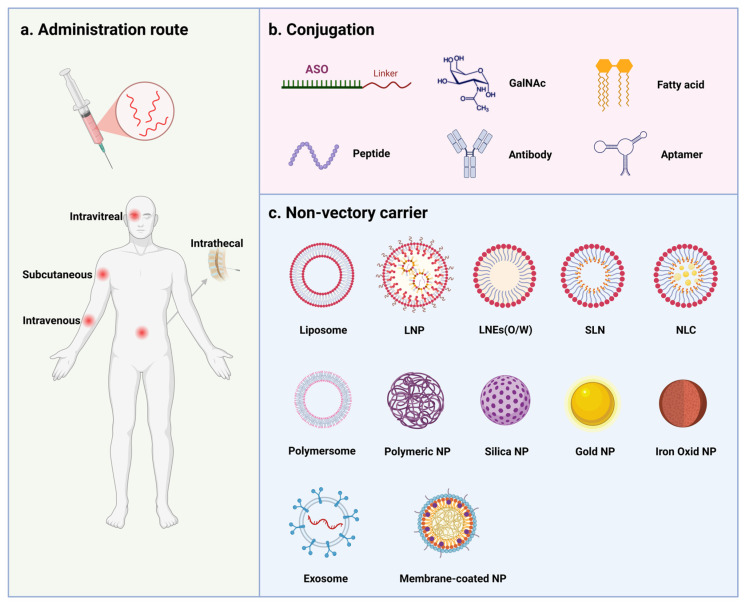
Delivery strategies for antisense oligonucleotides (ASOs). Major approaches to improving ASO delivery are shown, including (**a**) clinically used administration routes, such as intravitreal, intrathecal, subcutaneous, and intravenous delivery; (**b**) conjugate-based delivery, in which ASOs are linked to ligands or biomolecules (e.g., GalNAc, fatty acids, peptides, antibodies, or aptamers) to enhance tissue targeting and cellular uptake; and (**c**) non-viral carrier systems, including lipid-, polymer-, inorganic-, and extracellular vesicle-based platforms, which can protect ASOs, promote uptake, and improve intracellular delivery. Created in BioRender. Xu, A. (2026) https://BioRender.com/xplf0q4, accessed on 31 March 2026.

Despite chemical modifications, ASOs’ high hydrophilicity and large molecular weight limit intrinsic transmembrane permeability, making efficient, safe, and precise delivery a critical bottleneck in ASO developments. The common delivery strategies are shown in Figure 4.

### 5.1. Naked ASOs

Naked ASOs depend on their inherent physicochemical properties for in vivo distribution and cellular uptake [6]. They demonstrate efficacy in local administration but face challenges in systemic delivery. Local administration methods, such as intravitreal injection, are commonly used for ocular diseases, as evidenced by the use of fomivirsen (Vitravene) [83]. In contrast, early systemic administration strategies, including intravenous or subcutaneous routes, required high doses to achieve passive uptake by the reticuloendothelial system, leading to non-specific accumulation in the liver and kidneys and raising biosafety concerns, as seen with mipomersen (Kynamro) [84]. Furthermore, systemic administration is ineffective at penetrating brain tissue due to the blood–brain barrier [85]. However, ASOs exhibit a prolonged half-life in cerebrospinal fluid, lasting several months, which facilitates extensive distribution in the spinal cord and brain [73]. Consequently, intrathecal injection of naked ASOs has become the “gold standard” for treating central nervous system diseases, as exemplified by nusinersen (Spinraza) [86,87].

### 5.2. Conjugate-Based Delivery

Conjugate-based delivery attaches biologically active ligands or carriers to ASOs, thereby precisely modulating their delivery properties [33]. Ligands, such as carbohydrates, vitamins, and small molecules, are conjugated to the terminals of ASOs through click chemistry or linkers, exploiting receptor–ligand interactions to facilitate tissue-specific endocytosis [33,88]. For instance, trivalent GalNAc binds to the asialoglycoprotein receptor, enhancing hepatic targeting, reducing the required dosage, and extending dosing intervals [35]. Comparative studies between inotersen (Tegsedi) and GalNAc-conjugated eplontersen (Wainua), both targeting transthyretin, demonstrate that eplontersen exhibits superior efficacy and a lower incidence of thrombocytopenia and nephrotoxicity [29]. However, the hepatocyte-specific targeting of GalNAc presents limitations for certain therapeutic applications. For example, bepirovirsen (GSK3228836) [89,90], which is more effective than its GalNAc-conjugated counterpart (GSK3389404) [91,92], circumvents the use of GalNAc to facilitate entry into hepatocytes for viral transcript degradation and access to non-parenchymal cells for immune stimulation, thereby enhancing the treatment of hepatitis B [93].

Lipophilic moieties, such as cholesterol, palmitic acid, vitamin E, and bile acids, have been shown to enhance the pharmacokinetics and cellular uptake of ASOs [94]. Imetelstat (Rytelo), a telomerase inhibitor approved in 2024, utilizes a palmitoyl modification and a specialized phosphorothioamidate (N3′-P5′) backbone to improve exposure and uptake, thereby increasing its efficacy in treating bone marrow disease [95,96]. Studies have demonstrated that ASOs conjugated with fatty-acid can improve muscle cellular uptake and gene-silencing potency [97], while ASOs conjugated with vitamin E or cholesterol enhance tumor uptake and activity [98].

Biomacromolecules, including antibodies, peptides, and aptamers, facilitate “precise targeting” and enable penetration into deep tissues [99]. Representative examples include anti-transferrin receptor 1 (TfR1)-based antibody conjugates, which have been developed to enhance delivery across the blood–brain barrier and into muscle through receptor-mediated transcytosis and internalization [100,101]. Peptide–oligonucleotide conjugates (POCs) utilize cell-penetrating peptides or homing peptides to improve membrane translocation and facilitate endosomal escape, as demonstrated by peptide-conjugated PMO technology in the treatment of neuromuscular diseases [75,102]. Aptamers represent a complementary targeting strategy. They are short single-stranded nucleic acids that fold into defined three-dimensional structures and bind cell-surface proteins with high affinity and specificity [103]. In delivery systems, they function as targeting ligands that promote receptor-selective binding and internalization of oligonucleotide cargoes, thereby improving access to receptor-expressing cells in otherwise difficult-to-reach tissues [104]. As a proof-of-principle example, gold nanoparticles decorated with an α7/β1 integrin-targeting aptamer have been used to deliver microRNA-206 to muscle satellite cells and improve muscle regeneration in a mouse model of Duchenne muscular dystrophy [105]. Collectively, these examples illustrate how biomacromolecular ligands can support more selective delivery to otherwise difficult-to-access tissues.

### 5.3. Carrier-Based Delivery

In addition to conjugate-based strategies, various carrier-based delivery systems are being developed to address the challenges associated with the cellular uptake of ASOs.

Lipid-based nanoparticles, which include liposomes, lipid nanoparticles (LNPs), lipid nanoemulsions (LNEs), solid lipid nanoparticles (SLNs), and nanostructured lipid carriers (NLCs), are designed to navigate physiological barriers effectively [106]. Among these, lipid nanoparticles, characterized by anionizable lipid core, are recognized as the most efficient vehicles for the delivery of small nucleic acids [107]. Solid lipid nanoparticles and nanostructured lipid carriers provide advantages such as stability and sustained release [108], while lipid nanoemulsions are particularly suitable for the solubilization of lipophilic cargo in multimodal systems [109].

Synthetic polymers such as polyethyleneimine and polylactic-co-glycolic acid, along with natural polymers like chitosan and hyaluronic acid, as well as lipid–polymer hybrid nanoparticles, facilitate the formation of polyplexes with ASOs through electrostatic interactions, thereby safeguarding them from degradation [106,110]. Additionally, the incorporation of stimulus-responsive modifications in the polymer side-chains, such as pH- or redox-responsive motifs, can potentially be employed to enhance the compartment-specific release of ASOs within cells [110].

Inorganic nanocarriers, such as mesoporous silica nanoparticles, gold nanoparticles, silver nanoparticles, and iron oxide nanoparticles, exhibit controllable properties and surface modification capabilities [111]. Mesoporous silica nanoparticles are characterized by their ultra-high specific surface area and ordered mesoporous structures, which facilitate efficient drug loading and controlled release [112]. Gold and silver nanoparticles utilize metal–sulfur bonds or electrostatic interactions to achieve high-density loading, thereby enhancing resistance to nucleases [113]. For instance, functionalized ASO–gold nanoparticles have been used to inhibit pathogenic genes in drug-resistant bacteria, thereby restoring sensitivity to β-lactam antibiotics [114]. Iron oxide nanoparticles enable magnetically targeted delivery through external magnetic fields and serve as theranostic agents, particularly as contrast agents in magnetic resonance imaging [115]. Additionally, the surface engineering of inorganic carriers can synergistically improve cellular uptake. For instance, ASOTARI, which consists of glucose polymer-modified silica nanoparticles, is selectively internalized by bacteria via the bacterial-specific ABC sugar transporter pathway, facilitating targeted treatment of drug-resistant bacterial keratitis [116].

Biomimetic and cell-derived carriers exhibit low immunogenicity and exceptional ability to penetrate biological barriers, effectively delivering therapeutic agents by emulating endogenous biological transport mechanisms [117]. Among them, exosomes (more broadly, extracellular vesicles) are of particular interest because their endogenous membrane proteins and lipid composition can influence biodistribution, cellular uptake, and membrane interactions, making them promising carriers for nucleic acid delivery [118]. Exosomes are natural facilitators of intercellular communication. They possess distinctive surface proteins and lipid compositions that endow them with tissue-targeting capabilities and membrane fusion potential, thereby protecting ASOs from immune clearance and aiding in endosomal escape [119]. However, their clinical translation is still limited by vesicle heterogeneity, large-scale manufacturing, cargo-loading efficiency, and the lack of standardized characterization methods [120]. CDK-004 (exoASO-STAT6), an exosome-mediated ASO targeting STAT6 for the treatment of hepatocellular carcinoma, entered early clinical evaluation, but its development was later discontinued, perhaps due to the complexity of the delivery system, as well as concerns regarding efficacy and safety [121]. Cell-membrane vesicles, which are created by coating carriers with membrane components derived from erythrocytes, leukocytes, or tumor cells, provide a “camouflage effect” that extends the circulation time of ASOs. Additionally, they exploit the chemotactic properties of the source cells to achieve targeted enrichment at sites of inflammation or tumor, thereby enhancing biocompatibility and targeting precision [122].

## 6. Clinical Translation Landscape

### 6.1. Insights from Approved Drugs

ASOs have emerged as a promising class of sequence-specific nucleic acid therapeutics, with their clinical efficacy substantiated by an increasing number of approved drugs across diverse disease areas. This overview synthesizes their key clinical breakthroughs and technological advancements to underscore their expanding role in contemporary therapy (see Table 2).

The clinical translation of ASOs began with technological exploration and regulatory validation, a foundational stage exemplified by the approval of fomivirsen (Vitravene) during the early technological era. Administered via intravitreal injection, Fomivirsen circumvented systemic delivery risks, minimized whole-body exposure, and facilitated the monitoring of therapeutic efficacy [15]. Its success not only marked a pivotal breakthrough in the clinical application of ASOs, but also provided regulatory validation for sequence-specific nucleic acid therapeutics, thereby laying the groundwork for subsequent advancements towards systemic delivery, the next critical stage in ASO evolution.

Building on the regulatory and technological foundations established by fomivirsen, ASOs have progressed to systemic delivery, demonstrating significant advancements in the treatment of liver-related diseases. The liver’s intrinsic capacity for high oligonucleotide uptake, due to its role as a major source of circulating proteins and metabolic factors, facilitates effective systemic delivery of ASOs [123]. Mipomersen (Kynamro), the first systemically administered ASO targeting APOB-100 for homozygous familial hypercholesterolemia, established safety parameters for systemic ASO delivery through its market withdrawal [88]. In contrast, volanesorsen (Waylivra) and olezarsen (Tryngolza), both targeting the APOC3 pathway, illustrate advancements in the platform. Notably, olezarsen achieves a 50–60% reduction in triglycerides through monthly subcutaneous administration, while eliminating thrombocytopenia toxicity and negating the need for complex risk evaluation and mitigation strategies [28,124]. In the context of hereditary transthyretin-mediated amyloidosis, the successful approvals of inotersen (Tegsedi) and eplontersen (Wainua) have established ASOs as a foundational therapy for liver-derived diseases [125]. Overall, cardiovascular and metabolic disorders serve as a critical bridge for ASOs transitioning from orphan drugs to chronic disease therapeutics, paving the way for their exploration in complex areas like neurological disorders.

Building on their success in treating liver and metabolic diseases, ASOs have achieved clinical prominence in the realm of neurological disorders, an area characterized by significant unmet medical needs. This advancement is largely attributed to the sophisticated application of PMO technology and enhanced delivery methods [20]. PMO technology enhances ASO stability and target specificity in the central nervous system, enabling the approval of four ASOs—eteplirsen (Exondys 51), golodirsen (Vyondys 53), viltolarsen (Viltepso), and casimersen (Amondys 45)—for the treatment of Duchenne muscular dystrophy by modulating splicing to restore the reading frame or enhance functional transcripts [49]. Additionally, the use of intrathecal injection allows for the bypassing of the blood–brain barrier, enabling direct delivery to the central nervous system, with nusinersen (Spinraza) serving as a benchmark for central-nervous-system-targeted ASOs [50]. Beyond this, tofersen (Qalsody), approved for SOD1-associated amyotrophic lateral sclerosis based on reductions in neurofilament light chain rather than solely clinical survival, has established a new paradigm for the accelerated approval applicable to other disease areas, including oncology [30].

Leveraging technological advances and approval paradigms from neurological and metabolic diseases, ASOs have expanded into oncology with innovative applications that extend their druggable space beyond traditional RNA expression modulation. Imetelstat (Rytelo), the first oligonucleotide telomerase inhibitor approved for oncological use, addresses a critical unmet need in patients with lower-to-intermediate-risk, transfusion-dependent myelodysplastic syndromes [126]. Unlike classical PS-gapmer ASOs, which primarily focus on modulating RNA expression, imetelstat binds to the template region of the telomerase RNA component, directly inhibiting enzymatic activity. This action results in telomere shortening and apoptosis of malignant clones, classifying it as a sequence-specific RNA-targeting oligonucleotide [127]. More broadly, this example highlights the oncology potential of ASOs beyond transcript knockdown: they can engage difficult-to-treat RNA-dependent cancer vulnerabilities, including functional RNA–protein complexes, aberrant splice variants, and other disease-defining transcripts, and may be particularly valuable in biomarker-selected settings or rational combination strategies [128,129]. Its approval not only expands the ASO therapeutic landscape to include functional inhibition of RNA–protein complexes, but also paves the way for ASO-based cancer therapies, potentially extending their application to areas such as inflammation and immunotherapy.

In conjunction with their expansion into oncology, ASOs have made significant strides in the fields of inflammation and immunotherapy—representing the latest frontier in their clinical development. This progress is marked by a strategic transition from acute symptom control to long-acting prevention, thereby enhancing patient adherence and disease management. A notable example of this shift is donidalorsen (Dawnzera), which received approval in 2025 for the prophylaxis of hereditary angioedema in patients aged 12 years and older. Donidalorsen offers flexible subcutaneous dosing options (every 4 or 8 weeks) and reduces the frequency and severity of attacks by reducing plasma prekallikrein levels to inhibit the overactivation of bradykinin pathway [130,131]. Its approval underscores the role of ASOs in sustainable disease management and broadens their clinical application scenarios. Collectively, these advancements across metabolic, neurological, oncologic, and inflammatory diseases reflect the progressive evolution of ASO technology. This evolution raises important questions regarding the logical sequence of their clinical development, which will be analyzed in the subsequent section.

### 6.2. Lessons from Failed Attempts

The clinical translation of ASOs remains constrained by multiple scientific, clinical, and strategic challenges. Examining discontinued or deprioritized clinical-stage programs can therefore provide practical insights for future ASO development. To illustrate these challenges while maintaining readability, Table 3 summarizes clinical-stage ASOs that did not achieve successful clinical translation, grouped according to the dominant primary reason for discontinuation, and also lists representative, rather than exhaustive, clinical trial identifiers. Based on this framework, the following discussion focuses on selected representative cases to highlight the major barriers and the lessons they offer for subsequent research and development.

One major challenge in the clinical application of ASOs is the imbalance between risk and benefit, which can lead to the discontinuation of products if long-term systemic toxicity or unacceptable safety signals arise, making risk management impractical. For example, (Kynamro) carried a boxed warning and required risk evaluation and mitigation strategies due to the risks of elevated transaminases and hepatic steatosis risks, and it was ultimately withdrawn from the market in 2019, only five years after receiving approval, due to severe hepatotoxicity [26]. Similarly, vupanorsen, which targets ANGPTL3, exemplifies a mid-stage termination resulting from an unfavorable risk–benefit profile. Pfizer and Ionis discontinued the program in 2022 because the Phase IIb lipid-lowering efficacy was insufficient to counterbalance emerging concerns about hepatic steatosis [132]. Additionally, SRP-5051 (vesleteplirsen), designed to improve the uptake of PMOs in neuromuscular diseases through conjugation with an arginine-rich cell-penetrating peptide, demonstrated greater potency than first-generation PMOs. However, it led to severe hypomagnesemia and the renal tubular toxicity due to renal accumulation of the cationic peptide, prompting Sarepta to announce the discontinuation of exon 51-skipping therapy for Duchenne muscular dystrophy [133]. These cases highlight that ASOs are not inherently unsafe; rather, the therapeutic window is collectively influenced by factors such as the chemical backbone, sequence characteristics, dosage exposure, and the risk profile of the patient population. For example, the FDA’s 2024 guidance on “Clinical pharmacology considerations for the development of oligonucleotide therapeutics” emphasizes systematic assessment of immunogenicity, hepatic and renal impairments, and drug–drug interactions [134].

Another critical hurdle is the failure to establish meaningful efficacy endpoints, particularly in oncology and other complex systemic diseases. Factors such as tissue heterogeneity, compensatory pathways, and the contexts of combination therapy frequently impede the translation of single-target knockdown into meaningful clinical benefits, particularly as the standard of care continues to evolve [135]. In oncology, this challenge is further complicated by marked tumor heterogeneity, both within and between patients. Target dependence may vary across tumor subclones, metastatic sites, and disease stages, meaning that effective knockdown in one cellular population may not translate into durable tumor control at the whole-patient level [136]. In addition, adaptive or acquired resistance, including compensatory signaling and clonal selection under treatment pressure, may further attenuate the benefit of single-target inhibition [137,138]. Clinical trial designs must address three critical questions: “what is the incremental benefit?”, “who benefits?” and “how does it complement existing therapies?” Without clear answers to these questions, Phase III programs may be terminated due to negative primary endpoints or futility, even when early molecular signals are positive [139]. A notable example is custirsen (OGX-011), which targets Clusterin in prostate cancer. Phase III clinical trials evaluating custirsen in combination with docetaxel and prednisone for metastatic castration-resistant prostate cancer failed to demonstrate a benefit in overall survival, as was similarly observed in a subsequent study involving cabazitaxel [140,141]. Casimersen, which received approval based on the surrogate endpoint of increased dystrophin expression, reported that its post-marketing confirmatory Phase III trial (NCT02500381) failed to meet the primary clinical endpoints [142], which underscores the uncertainty of translating surrogate endpoints into long-term clinical benefits. Sarepta is currently engaged in discussions with the FDA regarding potential withdrawal or alternative supporting evidence of casimersen [143]. The principal challenge for ASOs in complex diseases may not be the binding to target RNA, but rather achieving adequate effective exposure and effect size to impact survival or remission endpoints. Therefore, current development strategies should prioritize biomarker-driven patient stratification, combination therapies, and innovations in delivery methods.

An often underappreciated yet equally significant challenge in therapeutic development is the discrepancy between the site of delivery and the effect at the clinical endpoint. Successful delivery to the target organ does not guarantee access to cellular compartments essential for clinical endpoints. For instance, sepofarsen, developed by ProQR for targeting CEP290 in Leber congenital amaurosis 10, failed to meet the primary endpoint of improved best-corrected visual acuity in a pivotal Phase II/III trial [144]. In-depth analysis revealed that although sepofarsen restored full-length CEP290 protein at the molecular level, the photoreceptor cell structures in patients with advanced disease stages had undergone irreversible degeneration, and simultaneously, the concentrations of the drug in the central macular fovea may have been insufficient [145]. Similarly, tominersen (RG6042), developed for Huntington’s disease and targeting the huntingtin protein, demonstrated reductions in cerebrospinal fluid huntingtin protein levels in Phase I/II trials [146]. However, the Phase III was terminated prematurely due to a lack of clinical benefit and adverse trends in the high-dose cohorts [147]. A critical factor in this outcome was tominersen’s non-selective knockdown of both mutant and wild-type huntingtin proteins, with the latter being essential for neuronal survival [148]. These failures indicate that even with effective administration routes, functional endpoints may be limited by disease stage, exposure variability, and irreversible tissue damage.

In addition to scientific and regulatory challenges, the limited commercial viability of ASOs can impede their clinical application, even when these drugs possess a robust mechanistic rationale and demonstrated efficacy. Changes in the therapeutic landscape, diminished patient demand, or the emergence of superior treatment modalities can render certain drugs clinically unnecessary. For instance, fomivirsen (Vitravene), a landmark in pharmaceutical history, was voluntarily withdrawn from the European market in 2002 due to “commercial reasons rather than safety concerns [149]”, primarily because of the advent of highly active antiretroviral therapy, which drastically reduced cytomegalovirus retinitis incidence [26]. The clinical application of ASOs is thus influenced not only by scientific and regulatory factors but also by epidemiology of diseases, the evolution of treatment landscapes, and commercial accessibility, particularly in infectious diseases, ophthalmology, and rare diseases.

### 6.3. Trends in the Clinical Pipeline

ASO applications are expanding across neurological, neuromuscular, ophthalmic, respiratory, renal, inflammatory–immune, infectious, and oncological indications. Here, only representative examples are discussed; a summary of investigational drugs currently in clinical trials is provided in Table 4.

Most Phase III programs focus on indications with well-established regulatory and clinical pathways, aligning with recent approvals (e.g., olezarsen, eplontersen, donidalorsen, tofersen, and imetelstat). These candidates target three main areas: (i) liver-derived targets in cardiovascular or metabolic diseases and immune–inflammatory disorders; (ii) neurological diseases amenable to intrathecal injection; and (iii) infectious diseases with clear virologic endpoints or attack-frequency outcomes.

Pelacarsen (TQJ230; targeting Lp[a]) represents the largest global ASO clinical trial to date, and its cardiovascular outcomes trial readout will determine ASOs’ potential to penetrate the mainstream cardiovascular pharmacotherapy market [150]. ION582 (BIIB121) and GTX-102 (Apazunersen) exemplify “gene activation” strategy, they target UBE3A-silencing regions to “unsilence” the paternal UBE3A gene to address the root cause of Angelman syndrome through shifting from “replacement” to “restorative” therapy [151,152]. Additionally, competition between bepirovirsen (GSK3228836) and AHB-137 underscores that the unique clinical value of dual mechanism (target transcript degradation plus immune activation) in complex immune microenvironments of hepatitis B therapy [153].

Phase II trials serve as the primary arena for ASO target validation and platform iteration. Trabedersen (AP 12009; OT-101), initially terminated in glioma setting due to insufficient clinical benefit [154], was repurposed as a potent TGF-β2 inhibitor following the identification of TGF-β as a key driver of PD-1 blockade resistance [155], and Oncotelic is now pursuing regulatory approval for OT-101 for the treatment of pancreatic and lung cancer [156]. In the neuromuscular diseases, WVE-N531 (Exon 53 skipping therapy) uses PN chemistry stereochemical modifications to optimize pharmacology, targeting muscle satellite cells to promote myofiber regeneration and achieving substantial dystrophin restoration without carriers [41]. Learning from Tominersen, WVE-003 targets the mHTT SNP3 locus for allele-selective degradation in HD, preserving wtHTT while silencing mutant protein, and is poised to initiate pivotal Phase III trials [146].

Early-stage trials feature diverse delivery formats and exploratory mechanisms. DYNE-251 (exon 51) from Dyne Therapeutics and AOC-1044 (exon 44) from Avidity use TfR1 antibody conjugation on PMO backbones for active muscle cell transport, with early data showing superior exon-skipping efficiency and protein restoration compared to unconjugated PMOs [157]. In oncology, BP1002 (L-Bcl-2) was developed by encapsulating Bcl-2 antisense sequences in neutral-charged liposomes (lipobilisome), completing dose escalation with preliminary efficacy signals [158]. For bacterial infections, ASOTARI uses a “Trojan horse” strategy via bacteria-specific ABC sugar transporters, improving gene-silencing efficiency and in vivo antibacterial activity for drug-resistant pathogens treatment [116]. In neurological disease, central nervous system indications are expanding to rare genetic disorders (e.g., Pelizaeus–Merzbacher disease, Creutzfeldt–Jakob disease, and epileptic encephalopathy), while for Alzheimer’s disease and amyotrophic lateral sclerosis, research focuses on mechanism refinement and delivery optimization [159].

Beyond clinical-stage candidates, ASOs hold significant translational potential in other disease areas, particularly antifungal therapy [6,160]. Studies have shown that 2′-O-Me and LNA-gapmer ASOs can inhibit Efg1, a *Candida albicans* virulence transcriptional factor, suppressing hyphal formation, biofilm formation and virulence in *Galleria mellonella* infection models [161]. Multi-target strategies targeting virulence pathway regulatory nodes (e.g., Ras1 and Rim101) have also emerged, with combined 2′-OMe ASOs enhancing hyphal formation control [162]. A recent study constructed a functionalized nanoconstruct (FTNx) to silence Fks1 (β-1,3-glucan synthase) and Chs3 (chitin synthase), key fungal cell-wall biosynthesis genes, achieving synergistic inhibition in vitro and improved survival in a murine-disseminated candidiasis model [163]. Despite challenges in fungal cell wall penetration, endocytosis, and intracellular transport, ASO therapy holds promise as an adjunct to traditional antifungal through multi-targeting, higher-affinity backbones, and enhanced delivery systems.

Due to the highly programmable nature of sequence design, ASOs are ideal for individualized precision therapy, enabling direct translation of genetic sequencing data into drug synthesis [164]. Milasen, a landmark in gene therapy and individualized medicine (N-of-1 trials), completed the entire process from diagnosis to dosing in one year, successfully correcting a rare splicing mutation and alleviating epileptic symptoms [165]. This breakthrough catalyzed the establishment of the n-Lorem Foundation, dedicated to developing therapies for ultra-rare diseases, and promoted regulatory reforms for adaptive approval and rapid-response mechanisms [166] (N-of-1 of ASO therapy initiated by the n-Lorem Foundation are summarized in Table 5). The evolution of the ASO field is driving a paradigm shift from a “one-size-fits-all” approach to personalized medicine.

## 7. Challenges and Perspectives

### 7.1. Challenges

Despite the commercial success of ASO therapeutics in treating specific diseases, their expansion to broader therapeutic indications remains hindered by multiple interconnected challenges. These bottlenecks primarily revolve around bioavailability limitations, safety threshold optimization, and the lag in clinical evaluation frameworks, all of which demand targeted innovations to unlock the full potential of ASO-based therapies [3,26].

Delivery efficiency persists as the primary rate-limiting factor for ASO efficacy. Although hepatocyte-targeted therapies with GalNAc conjugation have achieved substantial success, macromolecular nucleic acids still face formidable biological barriers in extrahepatic targeted diseases [88]. The blood–brain barrier remains a major obstacle for central nervous system indications, restricting effective brain tissue penetration despite advances in intrathecal delivery [85]. Additionally, low endosomal escape rates limit cytosolic or nuclear access, while microbial cell wall penetration poses unique challenges for anti-infective applications [36]. These delivery hurdles collectively result in suboptimal target engagement and require excessive dosing, exacerbating safety concerns.

Balancing potency and toxicity represent another critical challenge. Ultra-high-affinity chemistries (e.g., LNA, cEt) enhance target binding but simultaneously increase the risk of off-target hybridization with homologous transcripts, leading to unintended gene silencing or cellular dysfunction [20]. Chemical modifications can significantly improve ASO pharmacokinetics, while they are prone to induce off-target effects and adverse events, including thrombocytopenia, hepatorenal toxicity, and immune-inflammatory responses [6].

The disconnect between preclinical models, surrogate biomarkers, and clinical outcomes magnifies development risk. For example, in Duchenne muscular dystrophy, increased dystrophin expression—used as a surrogate endpoint for approval—has not consistently translated to functional improvements in long-term clinical trials [50,144]. This gap highlights the need for more predictive biomarkers and clinical evaluation frameworks that better align molecular effects with patient-centric outcomes (e.g., mobility, quality of life, survival) [167]. Additionally, the high cost of ASO development and manufacturing—exacerbated by the need for personalized or ultra-rare disease therapies—raises accessibility concerns, particularly for patients in resource-limited settings [166].

### 7.2. Future Directions

To address these challenges, future research will focus on three interconnected pillars: innovative delivery systems, precision engineering of ASO molecules, and refined clinical development strategies. Advancements in delivery technology will prioritize tissue-specific targeting and enhanced transmembrane transport. For central nervous system disorders, novel strategies—such as the antibody–oligonucleotide conjugates (AOCs) targeting blood–brain barrier transport receptors (e.g., TfR1) or stimulus-responsive nanocarriers—aim to improve brain parenchymal penetration and cellular uptake [160]. For non-hepatic peripheral tissues (e.g., muscle, kidney), peptide conjugation (e.g., CPPs) and biomimetic carriers (e.g., exosomes, cell membrane vesicles) offer promising avenues to overcome endosomal barriers and reduce off-target accumulation [118]. Small-molecule endosomal escape enhancers, which disrupt endosomal membranes without inducing cytotoxicity, are also being explored to boost intracellular ASO bioavailability [99].

Precision engineering of ASOs will focus on optimizing specificity, stability, and safety. Stereopure synthesis—controlling the chiral configuration of PS linkages—reduces product heterogeneity and non-specific protein interactions, thereby narrowing the therapeutic window [41]. Allele-selective ASOs, designed to target mutant transcripts while sparing wild-type alleles (e.g., WVE-003 for Huntington’s disease), mitigate on-target toxicity associated with non-selective gene silencing [148]. Additionally, next-generation chemical modifications (e.g., 2′-MCE, BNAP-AEO) aim to maintain high binding affinity while minimizing hepatotoxicity and immunogenicity, expanding the applicability of ASOs to chronic disease populations requiring long-term treatment [3,55].

Refined clinical development strategies will emphasize biomarker-driven patient stratification and adaptive trial designs. Integrating transcriptomic and genomic data will enable the identification of patient subgroups most likely to benefit from ASO therapy, reducing trial size and improving success rates [168]. For complex diseases (e.g., cancer, hepatitis B), combination therapies—pairing ASOs with immune checkpoint inhibitors, small molecules, or other nucleic acid therapeutics—will leverage synergistic mechanisms to overcome compensatory pathways and enhance therapeutic efficacy [13,135]. Furthermore, regulatory frameworks for personalized ASOs (e.g., N-of-1 trials for ultra-rare diseases) will continue to evolve, streamlining approval pathways while ensuring safety and efficacy.

As these innovations mature, ASO therapeutics are poised to evolve from a niche orphan drug platform to the “third pillar” of pharmacotherapy—complementing small molecules and biologics. The expansion of ASOs to common diseases (e.g., cardiovascular disorders, neurodegenerative diseases) will be driven by large-scale clinical trials (e.g., pelacarsen for Lp[a]-mediated atherosclerosis [150]) and the validation of dual-mechanism strategies (e.g., bepirovirsen for hepatitis B, combining transcript degradation and immune activation [91]). Additionally, the emergence of RNA-editing ASOs (AIMers) and gene activation strategies (e.g., for Angelman syndrome) will extend ASO applications beyond gene silencing to precise transcript correction and restoration, addressing the root cause of genetic diseases without altering genomic DNA [152,169]. In summary, while significant challenges remain, the continuous refinement of chemical modifications, delivery systems, and clinical trial designs will unlock the full therapeutic potential of ASOs. By addressing unmet medical needs across rare and common diseases, ASOs are positioned to transform the landscape of precision medicine and improve outcomes for countless patients worldwide.

## Figures and Tables

**Table 1:** Classification and functional summary of major chemical modifications used in ASOs.

Name	Full Name	First Reported (Approx.)	Main Purpose(s)
Backbone modifications			
PO	Phosphodiester	Native/1950s	Native anionic linkage; reference scaffold for ASOs
PS	Phosphorothioate	1967	Improve nuclease resistance, plasma protein binding, and in vivo half-life
BS	Boranophosphate	Early 1990s	Tune backbone electronics and stability while preserving antisense activity
MP	Methylphosphonate	1969	Increase nuclease resistance and reduce backbone charge
PTE	Phosphotriester	1970s	Mask backbone charge and improve membrane-related properties
PA	Phosphoramidate	1970s	Improve stability and tune charge/protein interactions
PS2	Phosphorodithioate	Early 1990s	Further enhance nuclease resistance and alter lipophilicity/protein binding
N3 phosphoramidate	N3′-phosphoramidate	Early 1990s	Increase affinity and nuclease resistance
3′-methylene phosphonate	3′-methylene phosphonate linkage	Mid-1990s	Replace the natural phosphate linkage with a metabolically stable surrogate
C3-amide	C3-amide linkage	Late 1990s	Increase metabolic stability and tune local backbone conformation
Formacetal	Formacetal linkage	Late 1980s	Provide a non-phosphorus internucleoside linkage with altered charge and flexibility
Thioformacetal	Thioformacetal linkage	Early 1990s	Increase linkage stability relative to formacetal analogs
MMI	Methylene(methylimino) linkage	Mid-1990s	Introduce an achiral non-phosphorus linkage with improved stability
PMO	Phosphorodiamidate morpholino oligomer	1985–1993	Provide extreme nuclease resistance and low protein binding for splice switching
TMO	Thiophosphoramidate morpholino oligomer	Early 1990s	Further increase morpholino-like stability and binding performance
PNA	Peptide nucleic acid	1991	Maximize hybridization affinity and resistance to nuclease/protease degradation
tcDNA	Tricyclo-DNA	Early 2000s	Increase affinity, nuclease resistance, and activity in splice-modulation settings
SNA	Serinol nucleic acid	2004	Provide a neutral/flexible scaffold with broad hybridization capability
Ribose modifications			
2′-O-Me	2′-O-methyl	1960s	Increase nuclease resistance and reduce innate immune stimulation
2′-O-MOE	2′-O-(2-methoxyethyl)	1995	Increase affinity, nuclease resistance, and tolerability
2′-F	2′-fluoro	1960s	Increase affinity and nuclease resistance with minimal steric bulk
LNA	Locked nucleic acid	1998	Strongly increase target affinity and shorten effective ASO length
cEt	Constrained ethyl	2010	Provide LNA-like affinity with an improved therapeutic index
UNA	Unlocked nucleic acid	2004	Increase local flexibility and tune duplex asymmetry
GNA	Glycol nucleic acid	1972	Alter sugar topology and base-pairing behavior
ENA	Ethylene-bridged nucleic acid	2001	Increase affinity and nuclease resistance through conformational preorganization
BNA(NC)	N-carbamoyl bridged nucleic acid	Late 2000s	Retain high affinity while improving safety/tissue-distribution properties
BNA(COC)	COC-type bridged nucleic acid	Early 2010s	Further tune affinity, rigidity, and in vivo profile
Base modifications			
5-MeC	5-methylcytosine	1950s	Reduce CpG/TLR9-driven innate immune activation while preserving base pairing
5-MeU	5-methyluridine	1950s	Tune stacking, duplex stability, and immune recognition
Ψ	Pseudouridine	1951	Increase RNA stability and reduce innate immune sensing
m1Ψ	N1-methylpseudouridine	1961	Further reduce innate sensing and improve translation efficiency

Notes: Representative chemical modifications used in antisense oligonucleotides (ASOs) are categorized as backbone, ribose, and base modifications. First Reported refers to the approximate time at which the corresponding modification scaffold or nucleoside was first described in the literature, rather than its first therapeutic or clinical application. Approximate time ranges are used where the literature contains multiple related early reports or where the modification was developed progressively from closely related precursor chemistries. Main Purpose(s) summarizes the principal rationale for introducing each modification, including improvements in nuclease resistance, target-binding affinity, pharmacokinetic behavior, tolerability, or immunological profile. The table is intended as a structured overview of major ASO-relevant chemical modifications, rather than an exhaustive inventory of all reported analogs.

**Table 2:** Approved ASOs and current market status.

Drug Name	Trade Name	First Approval	Company	Target	Indication	Mechanism	Modification	Delivery Roure	Status
Fomivirsen	Vitravene	1998	Ionis (Carlsbad, CA, USA) & Novartis (Basel, Switzerland)	CMV mRNA	CMV retinitis	RNase H mediated	PS	Naked/IVT	Withdrawn
Mipomersen	Kynamro	2013	Ionis (Carlsbad, CA, USA) & Genzyme (Sanofi; Cambridge, MA, USA)	ApoB-100	HoFH	RNase H mediated	2′-MOE Gapmer	Naked/SC	Withdrawn
Eteplirsen	Exondys 51	2016	Sarepta (Cambridge, MA, USA)	Dys Exon 51	DMD	Steric blocking	PMO	Naked/IV	Marketed
Nusinersen	Spinraza	2016	Ionis (Carlsbad, CA, USA) & Biogen (Cambridge, MA, USA)	SMN2	SMA	Steric blocking	2′-MOE, PS	Naked/IT	Marketed
Inotersen	Tegsedi	2018	Ionis (Carlsbad, CA, USA) & Sobi (Stockholm, Sweden)	TTR	hATTR Amyloidosis	RNase H mediated	2′-MOE Gapmer	Naked/SC	Marketed
Volanesorsen	Waylivra	2019	Ionis (Carlsbad, CA, USA) & Sobi (Stockholm, Sweden)	APOC3	FCS	RNase H mediated	2′-MOE Gapmer	Naked/SC	Marketed
Golodirsen	Vyondys 53	2019	Sarepta (Cambridge, MA, USA)	Dys Exon 53	DMD	Steric blocking	PMO	Naked/IV	Marketed
Viltolarsen	Viltepso	2020	Nippon Shinyaku (Kyoto, Japan)	Dys Exon 53	DMD	Steric blocking	PMO	Naked/IV	Marketed
Casimersen	Amondys 45	2021	Sarepta (Cambridge, MA, USA)	Dys Exon 45	DMD	Steric blocking	PMO	Naked/IV	Marketed
Tofersen	Qalsody	2023	Ionis (Carlsbad, CA, USA) & Biogen (Cambridge, MA, USA)	SOD1	ALS	RNase H mediated	2′-MOE Gapmer	Naked/IT	Marketed
Eplontersen	Wainua	2023	AstraZeneca (Cambridge, UK) & Ionis (Carlsbad, CA, USA)	TTR	hATTR Amyloidosis	RNase H mediated	2′-MOE Gapmer	GalNAc/SC	Marketed
Imetelstat	Rytelo	2024	Geron Corporation (Foster City, CA, USA)	Telomerase hTR	MDS	Telomerase inhibition	N3′-P5′ Thio	Lipid/IV	Marketed
Olezarsen	Tryngolza	2024	Ionis (Carlsbad, CA, USA)	APOC3	FCS	RNase H mediated	2′-MOE Gapmer	GalNAc/SC	Marketed
Donidalorsen	Dawnzera	2025	Ionis (Carlsbad, CA, USA) & Otsuka (Tokyo, Japan)	PKK	HAE	RNase H mediated	2′-MOE Gapmer	GalNAc/SC	Marketed

Notes: This table includes ASO therapeutics that have received regulatory approval in at least one jurisdiction. First Approval refers to the year of first regulatory approval, whereas Status indicates their current marketed or withdrawn status at the time of writing.

**Table 3:** Discontinued clinical-stage antisense oligonucleotide (ASO) programs.

Primary Reason	Drug Name/Code	Phase	Key Clinical Trial ID	Target	Indication	Mechanism	Delivery Route
Risk–Benefit Imbalance	Drisapersen (GSK2402968)	III	NCT01254019	DMD Exon 51	DMD	Steric blocking	Naked/SC
	Vupanorsen (ISIS 703802)	II	NCT04516291	ANGPTL3	HTG	RNase H-mediated	GalNAc/SC
	RG-101	II	EudraCT: 2013-002978-49	miR-122	HCV	Anti-miR	GalNAc/SC
	AEG35156 (GEM640)	II	NCT00882869	XIAP Mrna	HCC	RNase H-mediated	Naked/IV
	SRP-5051 (vesleteplirsen)	II	NCT04004065	DMD Exon 51	DMD	Steric blocking	CPP/IV
	ION-827359	II	NCT03647228	SCNN1A/B/G	CF	RNase H-mediated	Naked/INH
	ALG-020572	I	NCT05001022	All HBV RNAs	HBV	RNase H-mediated	GalNAc/SC
	ISIS 388626	I	NCT00836225	SGLT2	T2DM and obesity	RNase H-mediated	Naked/SC
Failure to Establish Efficacy Endpoints	Aprinocarsen (ISIS 3521)	III	NCT00017407	PKC-α	Solid tumors	RNase H-mediated	Naked/IV
	Custirsen (OGX-011)	III	NCT01188187	Clusterin	CRPC	RNase H-mediated	Naked/IV
	Oblimersen (G3139)	III	NCT00024440	BCL2	Bcl-2-positive malignancies	RNase H-mediated	Naked/IV
	Alicaforsen (ISIS 2302)	III	NCT00063830	ICAM-1	CD	RNase H-mediated	Naked/IV
	Mongersen (GED-0301)	III	NCT02596893	SMAD7	CD	RNase H-mediated	Coating/PO
	GSK3389404 (GalNAc-bepirovirsen)	II	NCT03020745	All HBV RNAs	HBV	RNase H-mediated	GalNAc/SC
	OGX-427	II	NCT01120470	Hsp27	Solid tumors	RNase H-mediated	Naked/IV
	Danvatirsen (AZD9150)	II	NCT02983578	STAT3	Solid tumors	RNase H-mediated	Naked/IV
	ISIS 5132 (CGP69846A)	II	NCT00002587	C-RAF-1	Solid tumors	RNase H-mediated	Naked/IV
	ISIS 2503	II	NCT00004193	HRAS	PC	RNase H-mediated	Naked/IV
	Apatorsen (OGX-427)	II	NCT01829113	Hsp27	Solid tumors	RNase H-mediated	Naked/IV
	PGN-EDO51	II	NCT06079736	DMD Exon 51	DMD	Steric blocking	CPP/IV
	Avicursen (ATL1102)	II	ACTRN12618000936203	CD49d	DMD	RNase H-mediated	Naked/SC
	WVE-120101	II	NCT03225833	mHTT SNP1	HD	RNase H-mediated	Naked/IT
	WVE-120102	II	NCT03225846	mHTT SNP1	HD	RNase H-mediated	Naked/IT
	AZD5312	I/II	NCT03300505	AR	CRPC	RNase H-mediated	Naked/IV
	BIIB105 (ION541)	I/II	NCT04494256	ATXN2	ALS (ATXN2)	RNase H-mediated	Naked/IT
	EZN-2968	I	NCT01120288	HIF-1α	Multiple cancers	RNase H-mediated	Naked/IV
	RO7070179 (rename of EZN-2968)	I	NCT02564614	HIF-1α	HCC	RNase H-mediated	Naked/IV
	RO7062931	I	NCT03038113	All HBV RNAs	HBV	RNase H-mediated	GalNAc/SC
	BIIB078 (IONIS-C9Rx)	I	NCT03626012	C9orf72	ALS/FTD	RNase H-mediated	Naked/IT
Mismatch Between Delivery Site and Endpoint Effect	Tominersen (RG6042)	III	NCT03761849 NCT02519036	HTT	HD	RNase H-mediated	Naked/IT
	Sepofarsen (QR-110)	III	NCT03913143 NCT03140969	CEP290	LCA10	Steric blocking	Naked/IVT
	WVE-004	I	NCT04931862 NCT05683860	C9orf72	ALS/FTD	RNase H-mediated	Naked/IT
Limited Commercial Viability	IONIS-DGAT2Rx	II	NCT03334214	DGAT2	HS	RNase H-mediated	Naked/SC
	IONIS-GHR-LRx	II	NCT04522180	GHR	Acromegaly	RNase H-mediated	GalNAc/SC
	Miravirs (SPC3649)	II	NCT01200420	miR-122	HCV	Anti-miR	Naked/SC
	QR-1123	II	NCT04123626	RHO P23H	adRP	RNase H-mediated	Naked/IVT
	Fesomersen (ISIS 416858)	II	NCT03358030	Factor XI	Thromboprophylaxis	RNase H-mediated	Naked/SC
	QR-313	II	NCT03605069	COL7A1 Exon73	RDEB	Steric blocking	Naked/TOP
	RG125 (AZD4076)	I/II	NCT02612662	miR-103/107	T2DM	Anti-miR	GalNAc/SC
Other Reasons	Trabedersen (AP 12009)	III	NCT00761280	TGF-β2	Glioma	RNase H-mediated	Naked/intratumoral perfusion
	ISIS-GCGRRx	II	NCT02824003	GCGR	T2DM	RNase H-mediated	Naked/SC
	ISIS-GCCRRx	II	NCT01968265	GCCR	T2DM	RNase H-mediated	Naked/SC
	ISIS-FGFR4Rx	II	NCT02476019	FGFR4	Obesity	RNase H-mediated	Naked/SC
	CIVI-007	II	NCT04164888	PCSK9	Hypercholesterolemia	RNase H-mediated	GalNAc/SC
	IONIS-PTP1BRx (ISIS-404173)	II	NCT01918865	PTP1B	T2DM	RNase H-mediated	Naked/SC
	Atesidorsen (ATL1103)	II	ACTRN12615000289516	GHR	Acromegaly	RNase H-mediated	Naked/SC
	G4460 (LR-3001)	II	NCT00002592	c-myb	CLL	RNase H-mediated	Naked/IV
	Gataparsen (ISIS-23722)	II	NCT01107444	BIRC5	Second-line NSCLC	RNase H-mediated	Naked/IV
	Cavrotolimod (AST-008)	I/II	NCT03684785	TLR9	PD-1-resistant tumors	Immune activation	Naked/SC
	CDK-004	I	NCT05375604	STAT6	HCC	RNase H-mediated	Exosome/IV
	Radavirsen (AVI-7100)	I	NCT01747148	M1/M2	Influenza A virus	Steric blocking	PMOplus/IV

Notes: Primary failure reason was assigned according to the dominant cause of discontinuation within the mechanistic framework of this review. In cases with overlapping contributing factors, each ASO was assigned to the single most dominant and mechanistically informative primary reason. (1) Risk and Benefit Imbalance refers to programs discontinued primarily because of safety/tolerability liabilities or an unfavorable benefit–risk profile; (2) Failure to Establish Efficacy Endpoints refers to programs that did not demonstrate clinically meaningful efficacy; (3) Mismatch Between Delivery Site and Endpoint Effect refers to programs in which target-organ delivery and/or molecular target engagement was observed but did not translate into the intended clinical outcome; (4) Limited Commercial Viability refers to discontinuation driven mainly by market, competitive, epidemiologic, or portfolio considerations; (5) Other Reasons includes programs with mixed, non-specific, or insufficiently disclosed causes. For categories such as Limited Commercial Viability and Other Reasons, trial registries provide clinical context but do not alone establish the discontinuation rationale; sponsor disclosures and public development updates were also considered. Only representative clinical trial identifiers are shown in the main table to improve readability; these identifiers are not exhaustive and were selected to capture the most relevant late-stage, decisive, or program-defining studies. Phase indicates the highest clinical stage reached before discontinuation, deprioritization, or program termination. Data were curated through January 2026.

**Table 4:** ASO candidates in ongoing clinical development.

Drug Name/Code	Key Trial ID(s)	Target	Indication	Mechanism	Modification	Delivery Route
Phase III						
Neurologic/Neuromuscular Disorders						
Zilganersen (ION373)	NCT04849741	GFAP	AxD	RNase H-mediated	2′-MOE gapmer	IT/naked
ION582 (BIIB121)	NCT06914609	UBE3A-ATS	AxD	RNase H-mediated	2′-MOE gapmer	IT/naked
GTX-102 (apazunersen)	NCT06617429	UBE3A-ATS	AxD	RNase H-mediated	2′-MOE gapmer	IT/naked
ION363 (jacifusen)	NCT04768972	FUS	ALS(FUS)	RNase H-mediated	2′-MOE gapmer	IT/naked
Zorevunersen (STK-001)	NCT06872125	SCN1A	DS	TANGO	2′-MOE ODN	IT/naked
Eteplirsen (approved LTE)	NCT02420379	DMD exon 51	DMD	Steric blocking	PMO	IV/naked
Infectious Diseases						
Bepirovirsen (GSK3228836)	NCT05630820	All HBV RNAs	HBV; CHB	RNase H-mediated and immune activation	2′-MOE gapmer	SC/naked
AHB-137	NCT07246889	All HBV RNAs	HBV	RNase H-mediated and immune activation	Med-Oligo^TM^	SC/naked
Ophthalmic Disorders						
NEXAGON (lufepirsen)	NCT05966493	Connexin 43	PCED	Steric blocking	ODN	Eye gel/naked
Immune/Renal/Hemostatic Disorders						
Sefaxersen (IONIS-FB-LRx)	NCT05797610	Complement factor B	IgA nephropathy	RNase H-mediated	2′-MOE gapmer	SC/GalNAc
Donidalorsen (approved LTE)	NCT05139810	PKK	HAE	RNase H-mediated	2′-MOE gapmer	SC/GalNAc
Cardiometabolic Disorders						
Pelacarsen (TQJ230)	NCT04023552	LPA	Lp(a), CVD	RNase H-mediated	2′-MOE gapmer	SC/GalNAc
Olezarsen	NCT05079919; NCT05355402; NCT05185843	APOC3	sHTG; HTG; FCS	RNase H-mediated	2′-MOE gapmer	SC/GalNAc
Eplontersen	NCT04136171	TTR	ATTR-CM	RNase H-mediated	2′-MOE gapmer	SC/GalNAc
Phase II						
Cardiometabolic Disorders						
AZD2693 (ION839)	NCT05809934	PNPLA3	MASH	RNase H-mediated	2′-MOE gapmer	SC/GalNAc
IONIS-AGT-LRx	NCT03714776	AGT	Resistant hypertension	RNase H-mediated	2′-MOE gapmer	SC/GalNAc
ION224 (IONIS-DGAT2Rx)	NCT03334214	DGAT2	MASH with fibrosis	RNase H-mediated	2′-MOE gapmer	SC/GalNAc
Ophthalmic Disorders						
QR-421a (ultevursen)	NCT06627179	USH2A exon 13	arRP	Steric blocking	2′-O-Me PS	IVT/naked
Immune/Renal/Hemostatic Disorders						
Fesomersen (BAY2976217)	NCT04534114	Factor XI	Thromboprophylaxis	RNase H-mediated	2′-MOE gapmer	SC/GalNAc
AZD2373 (opemalirsen)	NCT06824987	APOL1	AMKD	RNase H-mediated	2′-cEt gapmer	SC/naked
Oncology						
OT-101	NCT06079346	TGF-β2	PDAC; MPM	RNase H-mediated	PS	Intratumoral perfusion/naked
BP1001 (prexigebersen)	NCT02781883	Grb-2	AML; ALL; CML-BP; MDS	RNase H-mediated	P-ethoxy-DNA	IV/liposome
Danvatirsen (AZD9150)	NCT05814666	STAT3	HNSCC	RNase H-mediated	2′-cEt gapmer	IV/naked
Neurologic/Neuromuscular Disorders						
BIIB080 (IONIS-MAPT Rx)	NCT05399888	MAPT	AD	RNase H-mediated	2′-MOE gapmer	IT/naked
WVE-003	NCT05032196	mHTT SNP3	HD	RNase H-mediated	PN chemistry	IT/naked
WVE-N531	NCT04906460	Dystrophin exon 53	DMD	Steric blocking	PN chemistry	IV/naked
Phase I/II						
Neurologic/Neuromuscular Disorders						
Elsunersen (PRAX-222)	NCT05737784	SCN2A	DEE	RNase H-mediated	2′-MOE gapmer	IT/naked
DYNE-251	NCT05524883	Dystrophin exon 51	DMD	Steric blocking	PMO	IV/Fab-PMO
AOC-1044 (del-zota)	NCT05670730	Dystrophin exon 44	DMD	Steric blocking	PMO	IV/Fab-PMO
Ophthalmic Disorders						
ISTH0036	NCT02406833	TGF-β2	POAG	RNase H-mediated	LNA gapmer	IVT/naked
Phase I						
Ophthalmic Disorders						
ASOTARI	NCT06451172	Essential genes	Antibiotic-resistant bacterial keratitis	Trojan horse strategy	PNA	Eye drops/GP-SiNPs-asPNA
STK-002	ISRCTN41725621	OPA1	ADOA	TANGO	2′-MOE gapmer	IVT/naked
Neurologic/Neuromuscular Disorders						
NIO752	NCT05469360	TAU	AD	RNase H-mediated	2′-MOE gapmer	IT/naked
ION356	NCT05786433	PLP1	PMD	RNase H-mediated	2′-MOE/cEt gapmer	IT/naked
ION716	NCT06249918	Prion protein	CJD	RNase H-mediated	2′-MOE gapmer	IT/naked
AMX0114	NCT06665165	CAPN2	ALS (CAPN2)	RNase H-mediated	2′-MOE gapmer	IT/naked
Atipeksen	NCT07215416	ATM exon 53	A-T	Steric blocking	2′-MOE PS	IT/naked
Oncology						
BP1002 (Liposome)	NCT04072458	Bcl-2	Bcl-2-positive malignancies	RNase H-mediated	P-ethoxy-DNA	IV/Liposome
Danvatirsen (AZD9150)	NCT03819465; NCT05986240	STAT3	NSCLC; AML/MDS	RNase H-mediated	2′-cEt gapmer	IV/naked
OT-101 (rename of trabedersen)	NCT06579196	TGF-β2	NSCLC	RNase H-mediated	PS	Intratumoral perfusion/naked

Notes: Representative antisense oligonucleotide (ASO) candidates with ongoing clinical development are summarized. Programs are organized according to the highest active clinical trial stage identified at the time of data collection and are further grouped by broad disease area. Where the same ASO was evaluated in multiple studies within the same phase and shared the same target and platform, related indications were consolidated into a single row. Clinical trial identifiers are representative rather than exhaustive and were selected to anchor the most relevant ongoing or program-defining studies. Information was compiled from publicly available trial registries and sponsor disclosures through March 2026, and active development status was cross-checked against publicly available sponsor pipeline updates when necessary. Programs labeled “approved LTE” denote long-term extension studies or ongoing post-approval clinical follow-up.

**Table 5:** Patient-specific antisense ASOs developed for individualized therapy.

NCT Number	Target	Drug Name/Code	Indication	Mechanism	Sponsor	Status
NCT07197268	ASXL3	nL-ASXL3-001	BRS	RNase H-mediated	n-Lorem Foundation	Active
NCT07215146	ATM	ASO targeting ATM	A-T	Steric blocking	Academic institution	Active
NCT06706388	ATN1	nL-ATN1-002	DRPLA	RNase H-mediated	n-Lorem Foundation	Active
NCT07084311	ATN1	nL-ATN1-002	DRPLA	RNase H-mediated	n-Lorem Foundation	Active
NCT07221760	ATN1	nL-ATN1-001	DRPLA	RNase H-mediated	n-Lorem Foundation	Not yet recruiting
NCT06392126	CHCHD10	nL-CHCHD-001	ALS (CHCHD10)	RNase H-mediated	n-Lorem Foundation	Active
NCT06977451	CHCHD10	nL-CHCHD-001	ALS (CHCHD10)	RNase H-mediated	n-Lorem Foundation	Active
NCT07095686	CHCHD10	nL-CHCHD-001	ALS (CHCHD10)	RNase H-mediated	n-Lorem Foundation	Enrolling
NCT06565572	FLVCR1	nL-FLVC-001	PCARP	Steric blocking	Academic institution	Enrolling
NCT06816498	LMNB1	nL-LMNB1-001	ADLD	RNase H-mediated	n-Lorem Foundation	Active
NCT07197294	MAPK8IP3	nL-MAPK8-001	NEDBA	RNase H-mediated	n-Lorem Foundation	Active
NCT07177196	PRPH2	nL-PRPH2-001	RD	RNase H-mediated	n-Lorem Foundation	Active
NCT06314490	SCN2A	nL-SCN2A-002	SCN2A-related disorders	RNase H-mediated	Academic institution	Active
NCT07095712	TARDBP	nL-TARD-001	ALS (TDP-43)	RNase H-mediated	n-Lorem Foundation	Active
NCT07222371	TUBB4A	nL-TUBB4-001	Leukodystrophy	RNase H-mediated	Academic institution	Active

Notes: This table summarizes representative patient-specific antisense oligonucleotides developed for individualized therapy. Mechanism annotations were standardized to publicly disclosed program-level ASO strategies. Status refers to the current recruitment or activity status of the corresponding clinical study at the time of data collection.

## Data Availability

No new data were created or analyzed in this study. Data sharing is not applicable to this article.

## Abbreviations

The following abbreviations are used in this manuscript:

ASOs	Antisense oligonucleotides
mRNA	Messenger RNA
ncRNA	Non-coding RNA
siRNAs	Small interfering RNAs
miRNAs	MicroRNAs
saRNAs	Small activating RNAs
anti-miR	Anti-microRNA
PS	Phosphorothioate
FDA	Food and Drug Administration
2′-MOE	2′-O-methoxyethyl
2′-O-Me	2′-O-methyl
2′-cEt	2′-O-(2-cyanoethyl)
GalNAc	N-acetylgalactosamine
PMO	Phosphorodiamidate morpholino oligomer
PPMO	Peptide-conjugated PMO
PNA	Peptide nucleic acid
LNA	Locked nucleic acid
BNA	Bridged nucleic acid
Med-Oligo^TM^	Multi-segmented enhanced and dual-acting oligonucleotides
TANGO	Targeted augmentation of nuclear gene output
P-ethoxy-DNA	P-ethoxy deoxyribonucleic acid
N3′-P5′ Thio	N3′ → P5′ thio-phosphoramidate
IT	Intrathecal
IV	Intravenous
SC	Subcutaneous
IVT	Intravitreal
PO	Per Os
CMV retinitis	Cytomegalovirus retinitis
HoFH	Homozygous familial hypercholesterolemia
DMD	Duchenne muscular dystrophy
SMA	Spinal muscular atrophy
FCS	Familial chylomicronemia syndrome
ALS	Amyotrophic lateral sclerosis
MDS	Myelodysplastic syndrome
HAE	Hereditary angioedema
HTG	Hypertriglyceridemia
CF	Cystic fibrosis
HCV	Hepatitis C virus
HBV	Hepatitis B virus
T2DM	Type 2 diabetes mellitus
PC	Pancreatic cancer
CD	Crohn’s disease
CRPC	Castration-resistant prostate cancer
HD	Huntington’s disease
HS	Hepatic steatosis
HCC	Hepatocellular carcinoma
FTD	Frontotemporal dementia
LCA10	Leber congenital amaurosis type 10
adRP	Autosomal-dominant retinitis pigmentosa
RDEB	Recessive dystrophic epidermolysis bullosa
CLL	Chronic lymphocytic leukemia
NSCLC	Non-small-cell lung cancer
AxD	Alexander disease
DS	Dravet syndrome
CHB	Chronic hepatitis B
PCED	Posterior polymorphous corneal dystrophy
Lp(a)	Lipoprotein(a)
CVD	Cardiovascular disease
sHTG	Severe hypertriglyceridemia
ATTR-CM	Transthyretin amyloid cardiomyopathy
MASH	Metabolic dysfunction-associated steatohepatitis
AMKD	Acute megakaryoblastic leukemia
PDAC	Pancreatic ductal adenocarcinoma
MPM	Malignant pleural mesothelioma
AML	Acute myeloid leukemia
CML-BP	Chronic myeloid leukemia blast phase
HNSCC	Head and neck squamous cell carcinoma
AD	Alzheimer’s disease
DEE	Developmental and epileptic encephalopathy
POAG	Primary open-angle glaucoma
ADOA	Autosomal dominant optic atrophy
PMD	Pelizaeus–Merzbacher disease
CJD	Creutzfeldt–Jakob disease
A-T	Ataxia-telangiectasia
BRS	Bainbridge–Ropers syndrome
DRPL-A	Dentatorubral–pallidoluysian atrophy
PCARP	Posterior cortical atrophy with retinal pigmentary degeneration
ADLD	Adult-onset leukodystrophy
NEDBA	Neurodevelopmental disorder with brain atrophy
RD	Retinal dystrophy

## References

[1] Warner K.D., Hajdin C.E., Weeks K.M. (2018). Principles for targeting RNA with drug-like small molecules. Nat. Rev. Drug Discov..

[2] Cai Z., Ma H., Ye F., Lei D., Deng Z., Li Y., Gu R., Wen H. (2025). Discovery of RNA-Targeting Small Molecules: Challenges and Future Directions. MedComm.

[3] Liu M., Wang Y., Zhang Y., Hu D., Tang L., Zhou B., Yang L. (2025). Landscape of small nucleic acid therapeutics: Moving from the bench to the clinic as next-generation medicines. Signal Transduct. Target. Ther..

[4] Ruchi R., Raman G.M., Kumar V., Bahal R. (2025). Evolution of antisense oligonucleotides: Navigating nucleic acid chemistry and delivery challenges. Expert. Opin. Drug Discov..

[5] Seyhan A.A. (2024). Trials and Tribulations of MicroRNA Therapeutics. Int. J. Mol. Sci..

[6] Sun X., Setrerrahmane S., Li C., Hu J., Xu H. (2024). Nucleic acid drugs: Recent progress and future perspectives. Signal Transduct. Target. Ther..

[7] Santarpia G., Carnes E. (2024). Therapeutic Applications of Aptamers. Int. J. Mol. Sci..

[8] Ballantyne C.M., Vasas S., Azizad M., Clifton P., Rosenson R.S., Chang T., Melquist S., Zhou R., Mushin M., Leeper N.J. (2024). Plozasiran, an RNA Interference Agent Targeting APOC3, for Mixed Hyperlipidemia. N. Engl. J. Med..

[9] Mullard A. (2025). FDA approves anti-prekallikrein drug for hereditary angioedema. Nat. Rev. Drug Discov..

[10] Bennett C.F. (2019). Therapeutic Antisense Oligonucleotides Are Coming of Age. Annu. Rev. Med..

[11] Zamecnik P.C., Stephenson M.L. (1978). Inhibition of Rous sarcoma virus replication and cell transformation by a specific oligodeoxynucleotide. Proc. Natl. Acad. Sci. USA.

[12] Stephenson M.L., Zamecnik P.C. (1978). Inhibition of Rous sarcoma viral RNA translation by a specific oligodeoxyribonucleotide. Proc. Natl. Acad. Sci. USA.

[13] Crooke S.T., Vickers T.A., Liang X.H. (2020). Phosphorothioate modified oligonucleotide-protein interactions. Nucleic Acids Res..

[14] De Smet M.D., Meenken C.J., van den Horn G.J. (1999). Fomivirsen—A phosphorothioate oligonucleotide for the treatment of CMV retinitis. Ocul. Immunol. Inflamm..

[15] Geary R.S., Henry S.P., Grillone L.R. (2002). Fomivirsen: Clinical pharmacology and potential drug interactions. Clin. Pharmacokinet..

[16] Quemener A.M., Bachelot L., Forestier A., Donnou-Fournet E., Gilot D., Galibert M.D. (2020). The powerful world of antisense oligonucleotides: From bench to bedside. Wiley Interdiscip. Rev. RNA.

[17] Gagliardi M., Ashizawa A.T. (2021). The Challenges and Strategies of Antisense Oligonucleotide Drug Delivery. Biomedicines.

[18] Hagedorn P.H., Hansen B.R., Koch T., Lindow M. (2017). Managing the sequence-specificity of antisense oligonucleotides in drug discovery. Nucleic Acids Res..

[19] Crooke S.T., Liang X.-H., Baker B.F., Crooke R.M. (2021). Antisense technology: A review. J. Biol. Chem..

[20] Egli M., Manoharan M. (2023). Chemistry, structure and function of approved oligonucleotide therapeutics. Nucleic Acids Res..

[21] Crooke S.T. (2017). Molecular Mechanisms of Antisense Oligonucleotides. Nucleic Acid Ther..

[22] Hair P., Cameron F., McKeage K. (2013). Mipomersen sodium: First global approval. Drugs.

[23] Blom D.J., Raal F.J., Santos R.D., Marais A.D. (2019). Lomitapide and Mipomersen-Inhibiting Microsomal Triglyceride Transfer Protein (MTP) and apoB100 Synthesis. Curr. Atheroscler. Rep..

[24] Paunovska K., Loughrey D., Dahlman J.E. (2022). Drug delivery systems for RNA therapeutics. Nat. Rev. Genet..

[25] Obexer R., Nassir M., Moody E.R., Baran P.S., Lovelock S.L. (2024). Modern approaches to therapeutic oligonucleotide manufacturing. Science.

[26] Alhamadani F., Zhang K., Parikh R., Wu H., Rasmussen T.P., Bahal R., Zhong X.B., Manautou J.E. (2022). Adverse Drug Reactions and Toxicity of the Food and Drug Administration-Approved Antisense Oligonucleotide Drugs. Drug Metab. Dispos..

[27] Mercuri E., Darras B.T., Chiriboga C.A., Day J.W., Campbell C., Connolly A.M., Iannaccone S.T., Kirschner J., Kuntz N.L., Saito K. (2018). Nusinersen versus Sham Control in Later-Onset Spinal Muscular Atrophy. N. Engl. J. Med..

[28] Stroes E.S.G., Alexander V.J., Karwatowska-Prokopczuk E., Hegele R.A., Arca M., Ballantyne C.M., Soran H., Prohaska T.A., Xia S., Ginsberg H.N. (2024). Olezarsen, Acute Pancreatitis, and Familial Chylomicronemia Syndrome. N. Engl. J. Med..

[29] Coelho T., Marques W., Dasgupta N.R., Chao C.C., Parman Y., França M.C., Guo Y.C., Wixner J., Ro L.S., Calandra C.R. (2023). Eplontersen for Hereditary Transthyretin Amyloidosis with Polyneuropathy. JAMA.

[30] McGuigan A., Blair H.A. (2025). Tofersen: A Review in Amyotrophic Lateral Sclerosis Associated with SOD1 Mutations. CNS Drugs.

[31] Lennox A.L., Huang F., Behrs M.K., González-Sales M., Bhise N., Wan Y., Sun L., Berry T., Feller F., Morcos P.N. (2024). Imetelstat, a novel, first-in-class telomerase inhibitor: Mechanism of action, clinical, and translational science. Clin. Transl. Sci..

[32] Juliano R.L. (2016). The delivery of therapeutic oligonucleotides. Nucleic Acids Res..

[33] Kumar V., Turnbull W.B. (2023). Targeted delivery of oligonucleotides using multivalent protein-carbohydrate interactions. Chem. Soc. Rev..

[34] Miller C.M., Donner A.J., Blank E.E., Egger A.W., Kellar B.M., Østergaard M.E., Seth P.P., Harris E.N. (2016). Stabilin-1 and Stabilin-2 are specific receptors for the cellular internalization of phosphorothioate-modified antisense oligonucleotides (ASOs) in the liver. Nucleic Acids Res..

[35] Debacker A.J., Voutila J., Catley M., Blakey D., Habib N. (2020). Delivery of Oligonucleotides to the Liver with GalNAc: From Research to Registered Therapeutic Drug. Mol. Ther..

[36] Dowdy S.F. (2023). Endosomal escape of RNA therapeutics: How do we solve this rate-limiting problem?. RNA.

[37] Liang X.H., Sun H., Shen W., Crooke S.T. (2015). Identification and characterization of intracellular proteins that bind oligonucleotides with phosphorothioate linkages. Nucleic Acids Res..

[38] Lorenz P., Misteli T., Baker B.F., Bennett C.F., Spector D.L. (2000). Nucleocytoplasmic shuttling: A novel in vivo property of antisense phosphorothioate oligodeoxynucleotides. Nucleic Acids Res..

[39] Bäckström E., Bonetti A., Johnsson P., Öhlin S., Dahlén A., Andersson P., Andersson S., Gennemark P. (2024). Tissue pharmacokinetics of antisense oligonucleotides. Mol. Ther. Nucleic Acids.

[40] Le B.T., Chen S., Veedu R.N. (2024). Rational Design of Chimeric Antisense Oligonucleotides on a Mixed PO-PS Backbone for Splice-Switching Applications. Biomolecules.

[41] Iwamoto N., Butler D.C.D., Svrzikapa N., Mohapatra S., Zlatev I., Sah D.W.Y., Meena, Standley S.M., Lu G., Apponi L.H. (2017). Control of phosphorothioate stereochemistry substantially increases the efficacy of antisense oligonucleotides. Nat. Biotechnol..

[42] Geary R.S., Norris D., Yu R., Bennett C.F. (2015). Pharmacokinetics, biodistribution and cell uptake of antisense oligonucleotides. Adv. Drug Deliv. Rev..

[43] Vickers T.A., Wyatt J.R., Freier S.M. (2000). Effects of RNA secondary structure on cellular antisense activity. Nucleic Acids Res..

[44] Liang X.H., Sun H., Nichols J.G., Crooke S.T. (2017). RNase H1-Dependent Antisense Oligonucleotides Are Robustly Active in Directing RNA Cleavage in Both the Cytoplasm and the Nucleus. Mol. Ther..

[45] Rogalska M.E., Mancini E., Bonnal S., Gohr A., Dunyak B.M., Arecco N., Smith P.G., Vaillancourt F.H., Valcárcel J. (2024). Transcriptome-wide splicing network reveals specialized regulatory functions of the core spliceosome. Science.

[46] Terada C., Oh K., Tsubaki R., Chan B., Aibara N., Ohyama K., Shibata M.A., Wada T., Harada-Shiba M., Yamayoshi A. (2023). Dynamic and static control of the off-target interactions of antisense oligonucleotides using toehold chemistry. Nat. Commun..

[47] Shen X., Corey D.R. (2018). Chemistry, mechanism and clinical status of antisense oligonucleotides and duplex RNAs. Nucleic Acids Res..

[48] Scharner J., Aznarez I. (2021). Clinical Applications of Single-Stranded Oligonucleotides: Current Landscape of Approved and In-Development Therapeutics. Mol. Ther..

[49] Torres-Masjoan L., Aguti S., Zhou H., Muntoni F. (2025). Clinical applications of exon-skipping antisense oligonucleotides in neuromuscular diseases. Mol. Ther..

[50] Xu L., Irony I., Bryan W.W., Dunn B. (2017). Development of gene therapies-lessons from nusinersen. Gene Ther..

[51] Liang X.H., Shen W., Sun H., Migawa M.T., Vickers T.A., Crooke S.T. (2016). Translation efficiency of mRNAs is increased by antisense oligonucleotides targeting upstream open reading frames. Nat. Biotechnol..

[52] Merkle T., Merz S., Reautschnig P., Blaha A., Li Q., Vogel P., Wettengel J., Li J.B., Stafforst T. (2019). Precise RNA editing by recruiting endogenous ADARs with antisense oligonucleotides. Nat. Biotechnol..

[53] Genna V., Portella G., Sala A., Terrazas M., Serrano-Chacón I., González J., Villegas N., Mateo L., Castellazzi C., Labrador M. (2025). Systematic study of hybrid triplex topology and stability suggests a general triplex-mediated regulatory mechanism. Nucleic Acids Res..

[54] Vanderplow A.M., Dodis G.E., Rhee Y., Cikowski J.J., Gonzalez S., Smith M.L., Gogliotti R.G. (2024). Site-blocking antisense oligonucleotides as a mechanism to fine-tune MeCP2 expression. RNA.

[55] Bhamra J., Krishna M., Samaan G., Pattanayak S., Mukhopadhyay S. (2025). Toxicity of Antisense Oligonucleotides is Determined by the Synergistic Interplay of Chemical Modifications and Nucleotide Sequences, not by Either Factor Alone. Chembiochem.

[56] Hofman C.R., Corey D.R. (2024). Targeting RNA with synthetic oligonucleotides: Clinical success invites new challenges. Cell Chem. Biol..

[57] Kandasamy P., McClorey G., Shimizu M., Kothari N., Alam R., Iwamoto N., Kumarasamy J., Bommineni G.R., Bezigian A., Chivatakarn O. (2022). Control of backbone chemistry and chirality boost oligonucleotide splice switching activity. Nucleic Acids Res..

[58] Takahashi Y., Sato K., Wada T. (2022). Solid-Phase Synthesis of Boranophosphate/Phosphorothioate/Phosphate Chimeric Oligonucleotides and Their Potential as Antisense Oligonucleotides. J. Org. Chem..

[59] Patutina O.A., Gaponova Miroshnichenko S.K., Sen’kova A.V., Savin I.A., Gladkikh D.V., Burakova E.A., Fokina A.A., Maslov M.A., Shmendel E.V., Wood M.J.A. (2020). Mesyl phosphoramidate backbone modified antisense oligonucleotides targeting miR-21 with enhanced in vivo therapeutic potency. Proc. Natl. Acad. Sci. USA.

[60] Syed Y.Y. (2016). Eteplirsen: First Global Approval. Drugs.

[61] Le B.T., Paul S., Jastrzebska K., Langer H., Caruthers M.H., Veedu R.N. (2022). Thiomorpholino oligonucleotides as a robust class of next generation platforms for alternate mRNA splicing. Proc. Natl. Acad. Sci. USA.

[62] MacLelland V., Kravitz M., Gupta A. (2024). Therapeutic and diagnostic applications of antisense peptide nucleic acids. Mol. Ther. Nucleic Acids.

[63] Asanuma H., Kamiya Y., Kashida H., Murayama K. (2022). Xeno nucleic acids (XNAs) having non-ribose scaffolds with unique supramolecular properties. Chem. Commun..

[64] Murayama K., Yamano Y., Asanuma H. (2019). 8-Pyrenylvinyl Adenine Controls Reversible Duplex Formation between Serinol Nucleic Acid and RNA by [2 + 2] Photocycloaddition. J. Am. Chem. Soc..

[65] Yoshida T., Hagihara T., Uchida Y., Horiuchi Y., Sasaki K., Yamamoto T., Yamashita T., Goda Y., Saito Y., Yamaguchi T. (2024). Introduction of sugar-modified nucleotides into CpG-containing antisense oligonucleotides inhibits TLR9 activation. Sci. Rep..

[66] Crooke S.T., Baker B.F., Crooke R.M., Liang X.H. (2021). Antisense technology: An overview and prospectus. Nat. Rev. Drug Discov..

[67] Crooke S.T., Baker B.F., Witztum J.L., Kwoh T.J., Pham N.C., Salgado N., McEvoy B.W., Cheng W., Hughes S.G., Bhanot S. (2017). The Effects of 2′-O-Methoxyethyl Containing Antisense Oligonucleotides on Platelets in Human Clinical Trials. Nucleic Acid Ther..

[68] Abou Assi H., Rangadurai A.K., Shi H., Liu B., Clay M.C., Erharter K., Kreutz C., Holley C.L., Al-Hashimi H.M. (2020). 2′-O-Methylation can increase the abundance and lifetime of alternative RNA conformational states. Nucleic Acids Res..

[69] Sheng L., Rigo F., Bennett C.F., Krainer A.R., Hua Y. (2020). Comparison of the efficacy of MOE and PMO modifications of systemic antisense oligonucleotides in a severe SMA mouse model. Nucleic Acids Res..

[70] Masaki Y., Iriyama Y., Nakajima H., Kuroda Y., Kanaki T., Furukawa S., Sekine M., Seio K. (2018). Application of 2′-O-(2-N-Methylcarbamoylethyl) Nucleotides in RNase H-Dependent Antisense Oligonucleotides. Nucleic Acid Ther..

[71] Yamaguchi T., Komine H., Sugiura T., Kumagai R., Yoshida T., Sasaki K., Nakayama T., Kamada H., Inoue T., Obika S. (2025). Cycloalkane Incorporation Into the 2′,4′-Bridge of Locked Nucleic Acid: Enhancing Nuclease Stability, Reducing Phosphorothioate Modifications, and Lowering Hepatotoxicity in Antisense Oligonucleotides. JACS Au.

[72] Hagedorn P.H., Persson R., Funder E.D., Albæk N., Diemer S.L., Hansen D.J., Møller M.R., Papargyri N., Christiansen H., Hansen B.R. (2018). Locked nucleic acid: Modality, diversity, and drug discovery. Drug Discov. Today.

[73] Papargyri N., Pontoppidan M., Andersen M.R., Koch T., Hagedorn P.H. (2020). Chemical Diversity of Locked Nucleic Acid-Modified Antisense Oligonucleotides Allows Optimization of Pharmaceutical Properties. Mol. Ther. Nucleic Acids.

[74] Matsubayashi T., Yoshioka K., Lei Mon S.S., Katsuyama M., Jia C., Yamaguchi T., Hara R.I., Nagata T., Nakagawa O., Obika S. (2024). Favorable efficacy and reduced acute neurotoxicity by antisense oligonucleotides with 2′,4′-BNA/LNA with 9-(aminoethoxy)phenoxazine. Mol. Ther. Nucleic Acids.

[75] Haque U.S., Yokota T. (2023). Enhancing Antisense Oligonucleotide-Based Therapeutic Delivery with DG9, a Versatile Cell-Penetrating Peptide. Cells.

[76] Nan Y., Zhang Y.J. (2018). Antisense Phosphorodiamidate Morpholino Oligomers as Novel Antiviral Compounds. Front. Microbiol..

[77] Roberts T.C., Langer R., Wood M.J.A. (2020). Advances in oligonucleotide drug delivery. Nat. Rev. Drug Discov..

[78] Pollak A.J., Zhao L., Vickers T.A., Huggins I.J., Liang X.H., Crooke S.T. (2022). Insights into innate immune activation via PS-ASO-protein-TLR9 interactions. Nucleic Acids Res..

[79] He J., Seela F. (2002). Propynyl groups in duplex DNA: Stability of base pairs incorporating 7-substituted 8-aza-7-deazapurines or 5-substituted pyrimidines. Nucleic Acids Res..

[80] Gyi J.I., Gao D., Conn G.L., Trent J.O., Brown T., Lane A.N. (2003). The solution structure of a DNA*RNA duplex containing 5-propynyl U and C; comparison with 5-Me modifications. Nucleic Acids Res..

[81] Das A., Ghosh A., Kundu J., Egli M., Manoharan M., Sinha S. (2023). Synthesis and Biophysical Studies of High-Affinity Morpholino Oligomers Containing G-Clamp Analogs. J. Org. Chem..

[82] Fracchioni G., Vailati S., Grazioli M., Pirota V. (2024). Structural Unfolding of G-Quadruplexes: From Small Molecules to Antisense Strategies. Molecules.

[83] Aiello L.P., Brucker A.J., Chang S., Cunningham E.T., D’Amico D.J., Flynn H.W., Grillone L.R., Hutcherson S., Liebmann J.M., O’Brien T.P. (2004). Evolving guidelines for intravitreous injections. Retina.

[84] Migliorati J.M., Liu S., Liu A., Gogate A., Nair S., Bahal R., Rasmussen T.P., Manautou J.E., Zhong X.B. (2022). Absorption, Distribution, Metabolism, and Excretion of US Food and Drug Administration-Approved Antisense Oligonucleotide Drugs. Drug Metab. Dispos..

[85] Wu D., Chen Q., Chen X., Han F., Chen Z., Wang Y. (2023). The blood-brain barrier: Structure, regulation, and drug delivery. Signal Transduct. Target. Ther..

[86] Monine M., Norris D., Wang Y., Nestorov I. (2021). A physiologically-based pharmacokinetic model to describe antisense oligonucleotide distribution after intrathecal administration. J. Pharmacokinet. Pharmacodyn..

[87] Chiriboga C.A., Swoboda K.J., Darras B.T., Iannaccone S.T., Montes J., De Vivo D.C., Norris D.A., Bennett C.F., Bishop K.M. (2016). Results from a phase 1 study of nusinersen (ISIS-SMN(Rx)) in children with spinal muscular atrophy. Neurology.

[88] Anand P., Zhang Y., Patil S., Kaur K. (2025). Metabolic Stability and Targeted Delivery of Oligonucleotides: Advancing RNA Therapeutics Beyond The Liver. J. Med. Chem..

[89] Yuen M.F., Lim S.G., Plesniak R., Tsuji K., Janssen H.L.A., Pojoga C., Gadano A., Popescu C.P., Stepanova T., Asselah T. (2022). Efficacy and Safety of Bepirovirsen in Chronic Hepatitis B Infection. N. Engl. J. Med..

[90] Yuen M.F., Heo J., Jang J.W., Yoon J.H., Kweon Y.O., Park S.J., Tami Y., You S., Yates P., Tao Y. (2021). Safety, tolerability and antiviral activity of the antisense oligonucleotide bepirovirsen in patients with chronic hepatitis B: A phase 2 randomized controlled trial. Nat. Med..

[91] Vaillant A. (2023). Bepirovirsen/GSK3389404: Antisense or TLR9 agonists?. J. Hepatol..

[92] Yuen M.F., Heo J., Kumada H., Suzuki F., Suzuki Y., Xie Q., Jia J., Karino Y., Hou J., Chayama K. (2022). Phase IIa, randomised, double-blind study of GSK3389404 in patients with chronic hepatitis B on stable nucleos(t)ide therapy. J. Hepatol..

[93] Hui R.W., Mak L.Y., Fung J., Seto W.K., Yuen M.F. (2025). Prospect of emerging treatments for hepatitis B virus functional cure. Clin. Mol. Hepatol..

[94] Goodnow R.A. (2023). Reality check: Lipid-oligonucleotide conjugates for therapeutic applications. Expert. Opin. Drug Discov..

[95] Shahnoor S., Raza I.F., Rashid M., Panhwar H., Fatima I., Gul S., Nadeem K., Khan S., Mahmmoud Fadelallah Eljack M. (2025). FDA approval of imetelstat: A new era in the treatment of lower-risk myelodysplastic syndrome. Ann. Med. Surg..

[96] Platzbecker U., Santini V., Fenaux P., Sekeres M.A., Savona M.R., Madanat Y.F., Díez-Campelo M., Valcárcel D., Illmer T., Jonášová A. (2024). Imetelstat in patients with lower-risk myelodysplastic syndromes who have relapsed or are refractory to erythropoiesis-stimulating agents (IMerge): A multinational, randomised, double-blind, placebo-controlled, phase 3 trial. Lancet.

[97] Prakash T.P., Mullick A.E., Lee R.G., Yu J., Yeh S.T., Low A., Chappell A.E., Østergaard M.E., Murray S., Gaus H.J. (2019). Fatty acid conjugation enhances potency of antisense oligonucleotides in muscle. Nucleic Acids Res..

[98] Balachandran A.A., Poudel B.H., Rahimizadeh K., Chikkanna A., Veedu R.N. (2025). Enhancing the intracellular delivery of antisense oligonucleotides (ASO): A comparative study of aptamer, vitamin E, and cholesterol ASO conjugates. RSC Adv..

[99] Mangla P., Vicentini Q., Biscans A. (2023). Therapeutic Oligonucleotides: An Outlook on Chemical Strategies to Improve Endosomal Trafficking. Cells.

[100] Malecova B., Burke R.S., Cochran M., Hood M.D., Johns R., Kovach P.R., Doppalapudi V.R., Erdogan G., Arias J.D., Darimont B. (2023). Targeted tissue delivery of RNA therapeutics using antibody-oligonucleotide conjugates (AOCs). Nucleic Acids Res..

[101] Cochran M., Arias D., Burke R., Chu D., Erdogan G., Hood M., Kovach P., Kwon H.W., Chen Y., Moon M. (2024). Structure-Activity Relationship of Antibody-Oligonucleotide Conjugates: Evaluating Bioconjugation Strategies for Antibody-siRNA Conjugates for Drug Development. J. Med. Chem..

[102] Gushchina L.V., Vetter T.A., Frair E.C., Bradley A.J., Grounds K.M., Lay J.W., Huang N., Suhaiba A., Schnell F.J., Hanson G. (2022). Systemic PPMO-mediated dystrophin expression in the Dup2 mouse model of Duchenne muscular dystrophy. Mol. Ther. Nucleic Acids.

[103] Driscoll J., Gondaliya P., Zinn D.A., Jain R., Yan I.K., Dong H., Patel T. (2025). Using aptamers for targeted delivery of RNA therapies. Mol. Ther..

[104] Yang W., Ran C., Lian X., Wang Z., Du Z., Bing T., Zhang Y., Tan W. (2025). Aptamer-based targeted drug delivery and disease therapy in preclinical and clinical applications. Adv. Drug Deliv. Rev..

[105] Millozzi F., Milán-Rois P., Sett A., Delli Carpini G., De Bardi M., Gisbert-Garzarán M., Sandonà M., Rodríguez-Díaz C., Martínez-Mingo M., Pardo I. (2025). Aptamer-conjugated gold nanoparticles enable oligonucleotide delivery into muscle stem cells to promote regeneration of dystrophic muscles. Nat. Commun..

[106] Mehta M., Bui T.A., Yang X., Aksoy Y., Goldys E.M., Deng W. (2023). Lipid-Based Nanoparticles for Drug/Gene Delivery: An Overview of the Production Techniques and Difficulties Encountered in Their Industrial Development. ACS Mater. Au.

[107] Cullis P.R., Felgner P.L. (2024). The 60-year evolution of lipid nanoparticles for nucleic acid delivery. Nat. Rev. Drug Discov..

[108] Cai X., Dou R., Guo C., Tang J., Li X., Chen J., Zhang J. (2023). Cationic Polymers as Transfection Reagents for Nucleic Acid Delivery. Pharmaceutics.

[109] Borrajo M.L., Quijano A., Lapuhs P., Rodriguez-Perez A.I., Anthiya S., Labandeira-Garcia J.L., Valenzuela R., Alonso M.J. (2024). Ionizable nanoemulsions for RNA delivery into the central nervous system—Importance of diffusivity. J. Control Release.

[110] Takakusa H., Iwazaki N., Nishikawa M., Yoshida T., Obika S., Inoue T. (2023). Drug Metabolism and Pharmacokinetics of Antisense Oligonucleotide Therapeutics: Typical Profiles, Evaluation Approaches, and Points to Consider Compared with Small Molecule Drugs. Nucleic Acid Ther..

[111] Luther D.C., Huang R., Jeon T., Zhang X., Lee Y.W., Nagaraj H., Rotello V.M. (2020). Delivery of drugs, proteins, and nucleic acids using inorganic nanoparticles. Adv. Drug Deliv. Rev..

[112] Liu Y., Zhao M., Zhang M., Yang B., Qi Y.K., Fu Q. (2025). Mesoporous silica nanoparticle-based nanomedicine: Preparation, functional modification, and theranostic applications. Mater. Today Bio.

[113] Sharma A.R., Lee Y.H., Bat-Ulzii A., Bhattacharya M., Chakraborty C., Lee S.S. (2022). Recent advances of metal-based nanoparticles in nucleic acid delivery for therapeutic applications. J. Nanobiotechnol..

[114] Garza-Cardenas C.R., Leon-Buitimea A., Siller-Ceniceros A.A., Morones-Ramirez J.R. (2025). Antisense Oligonucleotide-Capped Gold Nanoparticles as a Potential Strategy for Tackling Antimicrobial Resistance. Microbiol. Res..

[115] Lapusan R., Borlan R., Focsan M. (2024). Advancing MRI with magnetic nanoparticles: A comprehensive review of translational research and clinical trials. Nanoscale Adv..

[116] Liu M., Chu B., Sun R., Ding J., Ye H., Yang Y., Wu Y., Shi H., Song B., He Y. (2023). Antisense Oligonucleotides Selectively Enter Human-Derived Antibiotic-Resistant Bacteria through Bacterial-Specific ATP-Binding Cassette Sugar Transporter. Adv. Mater..

[117] Xu G., Jin J., Fu Z., Wang G., Lei X., Xu J., Wang J. (2025). Extracellular vesicle-based drug overview: Research landscape, quality control and nonclinical evaluation strategies. Signal Transduct. Target. Ther..

[118] Herrmann I.K., Wood M.J.A., Fuhrmann G. (2021). Extracellular vesicles as a next-generation drug delivery platform. Nat. Nanotechnol..

[119] Zhang M., Hu S., Liu L., Dang P., Liu Y., Sun Z., Qiao B., Wang C. (2023). Engineered exosomes from different sources for cancer-targeted therapy. Signal Transduct. Target. Ther..

[120] Welsh J.A., Goberdhan D.C.I., O’Driscoll L., Buzas E.I., Blenkiron C., Bussolati B., Cai H., Di Vizio D., Driedonks T.A.P., Erdbrügger U. (2024). Minimal information for studies of extracellular vesicles (MISEV2023): From basic to advanced approaches. J. Extracell. Vesicles.

[121] Kamerkar S., Leng C., Burenkova O., Jang S.C., McCoy C., Zhang K., Dooley K., Kasera S., Zi T., Sisó S. (2022). Exosome-mediated genetic reprogramming of tumor-associated macrophages by exoASO-STAT6 leads to potent monotherapy antitumor activity. Sci. Adv..

[122] Liu L., Pan D., Chen S., Martikainen M.V., Kårlund A., Ke J., Pulkkinen H., Ruhanen H., Roponen M., Käkelä R. (2022). Systematic design of cell membrane coating to improve tumor targeting of nanoparticles. Nat. Commun..

[123] Gogate A., Belcourt J., Shah M., Wang A.Z., Frankel A., Kolmel H., Chalon M., Stephen P., Kolli A., Tawfik S.M. (2023). Targeting the Liver with Nucleic Acid Therapeutics for the Treatment of Systemic Diseases of Liver Origin. Pharmacol. Rev..

[124] Witztum J.L., Gaudet D., Freedman S.D., Alexander V.J., Digenio A., Williams K.R., Yang Q., Hughes S.G., Geary R.S., Arca M. (2019). Volanesorsen and Triglyceride Levels in Familial Chylomicronemia Syndrome. N. Engl. J. Med..

[125] De Michieli L., Lupi A., Sinigiani G., Tietto A., Salvalaggio A., Branca A., Da Pozzo S., Rizzo S., Cecchin D., Perazzolo Marra M. (2025). Pharmacological Management of Transthyretin Amyloid Cardiomyopathy: Where We Are and Where We Are Going. J. Clin. Med..

[126] Keam S.J. (2024). Imetelstat: First Approval. Drugs.

[127] Abaza Y., DeZern A.E. (2025). Imetelstat: A new addition to the therapeutic landscape of lower-risk MDS. Blood.

[128] DeFranciscis V., Amabile G., Kortylewski M. (2025). Clinical applications of oligonucleotides for cancer therapy. Mol. Ther..

[129] Çakan E., Lara O.D., Szymanowska A., Bayraktar E., Chavez-Reyes A., Lopez-Berestein G., Amero P., Rodriguez-Aguayo C. (2024). Therapeutic Antisense Oligonucleotides in Oncology: From Bench to Bedside. Cancers.

[130] Riedl M.A., Tachdjian R., Lumry W.R., Craig T., Karakaya G., Gelincik A., Stobiecki M., Jacobs J.S., Gokmen N.M., Reshef A. (2024). Efficacy and Safety of Donidalorsen for Hereditary Angioedema. N. Engl. J. Med..

[131] Syed Y.Y. (2025). Donidalorsen: First Approval. Drugs.

[132] Bergmark B.A., Marston N.A., Bramson C.R., Curto M., Ramos V., Jevne A., Kuder J.F., Park J.G., Murphy S.A., Verma S. (2022). Effect of Vupanorsen on Non-High-Density Lipoprotein Cholesterol Levels in Statin-Treated Patients with Elevated Cholesterol: TRANSLATE-TIMI 70. Circulation.

[133] Chwalenia K., Wood M.J.A., Roberts T.C. (2025). Progress and prospects in antisense oligonucleotide-mediated exon skipping therapies for Duchenne muscular dystrophy. J. Muscle Res. Cell Motil..

[134] Food and Drug Administration (FDA) Clinical Pharmacology Considerations for the Development of Oligonucleotide Therapeutics. https://www.fda.gov/regulatory-information/search-fda-guidance-documents/clinical-pharmacology-considerations-development-oligonucleotide-therapeutics.

[135] Emran T.B., Shahriar A., Mahmud A.R., Rahman T., Abir M.H., Siddiquee M.F., Ahmed H., Rahman N., Nainu F., Wahyudin E. (2022). Multidrug Resistance in Cancer: Understanding Molecular Mechanisms, Immunoprevention and Therapeutic Approaches. Front. Oncol..

[136] Frankell A.M., Dietzen M., Al Bakir M., Lim E.L., Karasaki T., Ward S., Veeriah S., Colliver E., Huebner A., Bunkum A. (2023). The evolution of lung cancer and impact of subclonal selection in TRACERx. Nature.

[137] Dagogo-Jack I., Shaw A.T. (2018). Tumour heterogeneity and resistance to cancer therapies. Nat. Rev. Clin. Oncol..

[138] Vasan N., Baselga J., Hyman D.M. (2019). A view on drug resistance in cancer. Nature.

[139] Nikanjam M., Kato S., Allen T., Sicklick J.K., Kurzrock R. (2025). Novel clinical trial designs emerging from the molecular reclassification of cancer. CA Cancer J. Clin..

[140] Chi K.N., Higano C.S., Blumenstein B., Ferrero J.M., Reeves J., Feyerabend S., Gravis G., Merseburger A.S., Stenzl A., Bergman A.M. (2017). Custirsen in combination with docetaxel and prednisone for patients with metastatic castration-resistant prostate cancer (SYNERGY trial): A phase 3, multicentre, open-label, randomised trial. Lancet Oncol..

[141] Beer T.M., Hotte S.J., Saad F., Alekseev B., Matveev V., Fléchon A., Gravis G., Joly F., Chi K.N., Malik Z. (2017). Custirsen (OGX-011) combined with cabazitaxel and prednisone versus cabazitaxel and prednisone alone in patients with metastatic castration-resistant prostate cancer previously treated with docetaxel (AFFINITY): A randomised, open-label, international, phase 3 trial. Lancet Oncol..

[142] Clinicaltrials.gov Study of SRP-4045 (Casimersen) and SRP-4053 (Golodirsen) in Participants with Duchenne Muscular Dystrophy (DMD) (ESSENCE). https://clinicaltrials.gov/study/NCT02500381.

[143] Parent Project Muscular Dystrophy. Sarepta Announces Completion of ESSENCE Trial for Exon 45 & 53 Skipping Therapies. https://www.parentprojectmd.org/sarepta-announces-completion-of-essence-trial-for-exon-45-53-skipping-therapies..

[144] Clinicaltrials.gov A Study to Evaluate Efficacy, Safety, Tolerability and Exposure After a Repeat-dose of Sepofarsen (QR-110) in LCA10 (ILLUMINATE) (ILLUMINATE). https://clinicaltrials.gov/study/NCT03913143.

[145] Russell S.R., Drack A.V., Cideciyan A.V., Jacobson S.G., Leroy B.P., Van Cauwenbergh C., Ho A.C., Dumitrescu A.V., Han I.C., Martin M. (2022). Intravitreal antisense oligonucleotide sepofarsen in Leber congenital amaurosis type 10: A phase 1b/2 trial. Nat. Med..

[146] Saade J., Mestre T.A. (2024). Huntington’s Disease: Latest Frontiers in Therapeutics. Curr. Neurol. Neurosci. Rep..

[147] McColgan P., Thobhani A., Boak L., Schobel S.A., Nicotra A., Palermo G., Trundell D., Zhou J., Schlegel V., Sanwald Ducray P. (2023). Tominersen in Adults with Manifest Huntington’s Disease. N. Engl. J. Med..

[148] Yao J.Y., Liu T., Hu X.R., Sheng H., Chen Z.H., Zhao H.Y., Li X.J., Wang Y., Hao L. (2024). An insight into allele-selective approaches to lowering mutant huntingtin protein for Huntington’s disease treatment. Biomed. Pharmacother..

[149] European Medicines Agency (EMA) Vitravene. https://www.ema.europa.eu/en/medicines/human/EPAR/vitravene.

[150] Clinicaltrials.gov Assessing the Impact of Lipoprotein (a) Lowering with Pelacarsen (TQJ230) on Major Cardiovascular Events in Patients with CVD (Lp(a)HORIZON). https://clinicaltrials.gov/study/NCT04023552.

[151] ClinicalTrials.gov Phase 3 Efficacy and Safety Study of GTX-102 in Pediatric Subjects with Angelman Syndrome (AS) (Aspire). https://clinicaltrials.gov/study/NCT06617429.

[152] Milazzo C., Narayanan R., Badillo S., Wang S., Almand R., Monshouwer R., Tzouros M., Golling S., Mientjes E., Chamberlain S. (2025). UBE3A reinstatement restores behaviorand proteome in an Angelman syndrome mouse model of imprinting defects. Mol. Autism.

[153] ClinicalTrials.gov Study of Bepirovirsen in Nucleos(t)Ide Analogue-treated Participants with Chronic Hepatitis B (B-Well 2) (B-Well 2). https://clinicaltrials.gov/study/NCT05630820.

[154] Jaschinski F., Rothhammer T., Jachimczak P., Seitz C., Schneider A., Schlingensiepen K.H. (2011). The antisense oligonucleotide trabedersen (AP 12009) for the targeted inhibition of TGF-β2. Curr. Pharm. Biotechnol..

[155] Mariathasan S., Turley S.J., Nickles D., Castiglioni A., Yuen K., Wang Y., Kadel E.E., Koeppen H., Astarita J.L., Cubas R. (2018). TGFβ attenuates tumour response to PD-L1 blockade by contributing to exclusion of T cells. Nature.

[156] ClinicalTrials.gov Trabedersen (OT-101) with Pembrolizumab for Newly Diagnosed Advanced NSCLC and Positive PD-L1. https://clinicaltrials.gov/study/NCT06579196.

[157] Li M., An H., Zhang J., Li W., Yu C., Wang L. (2025). Advances in the pharmaceutical development of antibody-oligonucleotide conjugates. Eur. J. Pharm. Sci..

[158] Kovecses O., Mercier F.E., McKeague M. (2024). Nucleic acid therapeutics as differentiation agents for myeloid leukemias. Leukemia.

[159] Bhati V., Prasad S., Kabra A. (2025). RNA-based therapies for neurodegenerative disease: Targeting molecular mechanisms for disease modification. Mol. Cell. Neurosci..

[160] Bruch A., Kelani A.A., Blango M.G. (2022). RNA-based therapeutics to treat human fungal infections. Trends Microbiol..

[161] Araújo D., Mil-Homens D., Henriques M., Silva S. (2022). Anti-EFG1 2′-OMethylRNA oligomer inhibits Candida albicans filamentation and attenuates the candidiasis in Galleria mellonella. Mol. Ther. Nucleic Acids.

[162] Barbosa A., Araújo D., Henriques M., Silva S. (2021). The combined application of the anti-RAS1 and anti-RIM101 2′-OMethylRNA oligomers enhances Candida albicans filamentation control. Med. Mycol..

[163] Chung J.Y., Hong Y.K., Jeon E., Yang S., Park A., Weissleder R., Chong Y.P., Chung H.J. (2025). Effective treatment of systemic candidiasis by synergistic targeting of cell wall synthesis. Nat. Commun..

[164] Carstens P.R., Yokota T. (2026). From Genomic Diagnosis to Personalized RNA Medicine: Advances in Next-Generation Sequencing and N-of-1 Antisense Oligonucleotide Therapies for Rare Genetic Diseases. Genes.

[165] Wilton-Clark H., Yan E., Yokota T. (2025). Milasen: The Emerging Era of Patient-Customized N-of-1 Antisense Oligonucleotides as Therapeutic Agents for Genetic Diseases. Methods Mol. Biol..

[166] Aartsma-Rus A., Garanto A., van Roon-Mom W., McConnell E.M., Suslovitch V., Yan W.X., Watts J.K., Yu T.W. (2023). Consensus Guidelines for the Design and In Vitro Preclinical Efficacy Testing N-of-1 Exon Skipping Antisense Oligonucleotides. Nucleic Acid Ther..

[167] Wu Y.F., Chen J.A., Jong Y.J. (2025). Treating neuromuscular diseases: Unveiling gene therapy breakthroughs and pioneering future applications. J. Biomed. Sci..

[168] Kernohan K.D., Boycott K.M. (2024). The expanding diagnostic toolbox for rare genetic diseases. Nat. Rev. Genet..

[169] Booth B.J., Nourreddine S., Katrekar D., Savva Y., Bose D., Long T.J., Huss D.J., Mali P. (2023). RNA editing: Expanding the potential of RNA therapeutics. Mol. Ther..

